# Benchmarking Generative AI Protein Models Reveals Differences Between Structural and Sequence-based Approaches

**DOI:** 10.1093/gpbjnl/qzag014

**Published:** 2026-02-15

**Authors:** Alexander J Barnett, Rajendra KC, Pratikshya Pandey, Pamodha Somasiri, Kirsten A Fairfax, Sandy Hung, Alex W Hewitt

**Affiliations:** Menzies Institute for Medical Research, University of Tasmania, Hobart, TAS 7000, Australia; Menzies Institute for Medical Research, University of Tasmania, Hobart, TAS 7000, Australia; Alphinia Bio, Hobart, TAS 7008, Australia; Menzies Institute for Medical Research, University of Tasmania, Hobart, TAS 7000, Australia; Alphinia Bio, Hobart, TAS 7008, Australia; School of Medicine, University of Tasmania, Hobart, TAS 7000, Australia; School of Medicine, University of Tasmania, Hobart, TAS 7000, Australia; Centre for Eye Research Australia, University of Melbourne, Melbourne, VIC 3002, Australia; Menzies Institute for Medical Research, University of Tasmania, Hobart, TAS 7000, Australia; School of Medicine, University of Tasmania, Hobart, TAS 7000, Australia; Centre for Eye Research Australia, University of Melbourne, Melbourne, VIC 3002, Australia

**Keywords:** Protein, Artificial intelligence, Generative artificial intelligence, Protease, Benchmark

## Abstract

Recent advances in artificial intelligence have led to the development of generative models for *de novo* protein design. In this study, we compared 13 state-of-the-art generative protein models, assessing their ability to produce feasible, diverse, and novel protein monomers. Structural diffusion models generally create designs with higher confidence in predicted structures and more biologically plausible energy distributions, but exhibit limited diversity and strong sequence biases. Conversely, protein language models generate more diverse and novel designs but with lower structural confidence. We also evaluated the ability of these models to generate unique proteins, conditionally based on the tobacco etch virus (TEV) protease. Generative models are successful in producing functional enzymes, albeit with diminished activity compared to the wild-type TEV. Our systematic benchmarking provides a foundation for evaluating and selecting generative protein models, while highlighting the complementary strengths of different generative paradigms. This framework will facilitate informed application of these tools for biomedical engineering and design.

## Graphical abstract

**Graphical Abstract:**
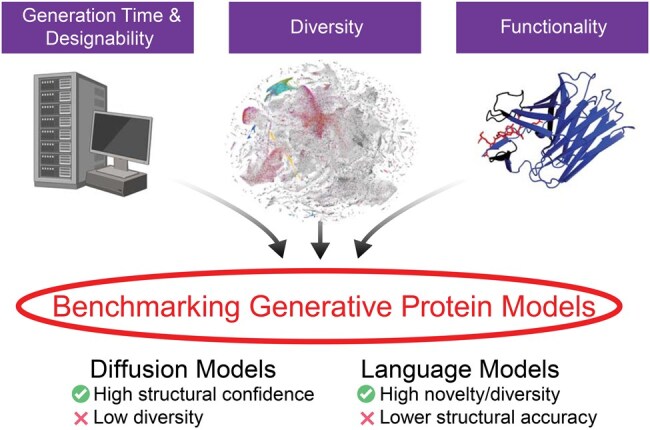

## Introduction

The remarkable versatility displayed by proteins in biological systems makes them an obvious class of interest for *de novo* molecular design. Protein functional properties are largely the product of their ordered tertiary structures, which emerge through folding of the cognate amino acid chain. Advances in deep learning have driven rapid performance gains in the prediction of protein structures and functional properties from arbitrary amino acid sequences [1,2]. Often outperforming traditional biophysical approaches, applications such as AlphaFold [3–5] highlight the capacity of data-driven deep learning models to attain fundamental insights into the relationships between protein sequence, structure, and function. Although predictive models can efficiently facilitate the assessment of candidate protein sequences, the space of possible sequences is vast, making the ability to appropriately prioritise or sample new proteins essential.

Seeking to leverage the understanding of protein biochemistry uncovered by predictive artificial intelligence (AI) models, a range of deep generative models have recently been trained on various empirical protein data, including sequences [6–10], backbone conformations [11–15], and full tertiary structures [16–18]. During training, these models learn a probability distribution over polypeptide space, which reflects both the physical constraints imposed by three-dimensional structures and the evolutionary pressures present in biological systems. Sampling from this distribution distils polypeptides to a more tractable candidate space enriched for biologically relevant proteins, and a subset of models can impose specific constraints during training or inference to refine the candidate space to one enriched for particular functional properties. However, it is currently not clear which generative models perform best.

Evaluating the capabilities of such generative models is a complex task, and little comparative work has been undertaken to systematically assess the merits and shortcomings of different approaches. While it is a reasonable assumption that a model trained on experimentally resolved structures would receive a higher concentration of exploitable information, it is unclear whether this is sufficient to offset the considerably lower volume of data compared to the abundance of known protein sequences. Until recently, most generative models of protein structures limited themselves to the task of generating candidate backbone conformations, relying on downstream models for sequence design and structural prediction. While more efficient during training, the extent to which the omission of side-chain influences during generation impacts the quality, diversity, and novelty of their designs is not well understood.

Herein, we compared the ability of 13 generative protein models spanning multiple paradigms (**Table 1**) to efficiently generate feasible, diverse, and novel protein monomers. We further applied five of these models to the relatively complex task of designing a monomer around a set of functional motifs from the tobacco etch virus (TEV) protease to assess their ability to generate proteins subject to specific constraints.

**Table 1:** Overview of generative protein models included in this study

Model	Paradigm	Output	Training data source					Postprocessing required for all-atom designs			Ability for TEV protease conditional design
			RCSB PDB	SCOPe	CATH	UniRef	BFD	Backbone refinement (Rosetta MinMover)	Sequence design (ProteinMPNN)	Structure prediction (OmegaFold)	
RFdiffusion [11]	D(Str)	BC	✔						✔	✔	✔
Genie [12]	D(Str)	BC (C*α*)		✔					✔	✔	
ProteinSGM [13]	D(Str)	BC			✔			✔	✔	✔	
FoldingDiff [14]	D(Str)	BC			✔				✔	✔	
FrameDiff [15]	D(Str)	BC	✔						✔	✔	
Chroma [16]	D(Str)	FTS	✔								✔
Protpardelle [17]	D(Str)	FTS			✔				*		✔
ProteinGenerator [18]	D(Seq)	FTS	✔							**	✔
EvoDiff [6]	D(Seq)	Sequences				✔				✔	✔
RITA [7]	APLM	Sequences				✔				✔	
ProGen2 [8]	APLM	Sequences				✔	✔			✔	
ProtGPT2 [9]	APLM	Sequences				✔				✔	
ESM-Design [10]	MTPLM	Sequences				✔				✔	

*Note*: D(Str), Diffusion (Structure); D(Seq), Diffusion (Sequence); APLM, Autoregressive Protein Language Model; MTPLM, Masked Token Protein Language Model; BC, backbone conformation; BC (C*α*), backbone conformation - C*α* coordinate only; FTS, full tertiary structure; TEV, tobacco etch virus. *, Protpardelle uses ProteinMPNN for sequence design at each denoising step of its structure generation. **, ProteinGenerator is adapted from the structural prediction network RoseTTAFold to generate sequence–structure pairs from noised protein sequences.

## Results

### Unconditional monomer generation

#### Implementation on Google Colaboratory

To assess the capabilities and usability of the selected models with a computational power accessible for open and economical use, we implemented each on the Google Colaboratory (Colab) platform according to their standard installation and execution instructions. RFdiffusion [11] and Chroma [16] provide existing public implementations on this platform, while RITA [7] and ProtGPT2 [9] are both easily accessed through the HuggingFace Transformers library. Most other models were straightforward to implement, requiring at most minor configuration changes and management of Python environments to successfully perform free generation. However, ProteinSGM [13] required substantial work to implement on Colab due to its dual-use CPU and GPU processing and errors encountered when executing as described in the documentation. The majority of models were capable of generating monomers within a length of 14 to 200 residues, and of performing batched generations at a set length. Exceptions were the autoregressive protein language models (RITA [7], ProGen2 [8], and ProtGPT2 [9]), which can only be supplied with a non-specific maximum generation length; FoldingDiff [14] and ProteinSGM [13], which have limited output length ranges (≤ 128 and 40–128 residues, respectively); and ESM-Design [10] and Genie [12], which experienced issues with GPU memory overflow when running batched generations on Colab. We found that ESM-Design with the default number of iterations would take ∼ 26 h to generate a single monomer of 200 residues on Colab. To include it in this study, we significantly decreased the number of iterations to make its generation time comparable to other protein language models. Rosetta FastDesign and FastRelax were disabled in the backbone refinement step of ProteinSGM [13] for similar reasons.

#### Generation time and designability

**Figure 1:**
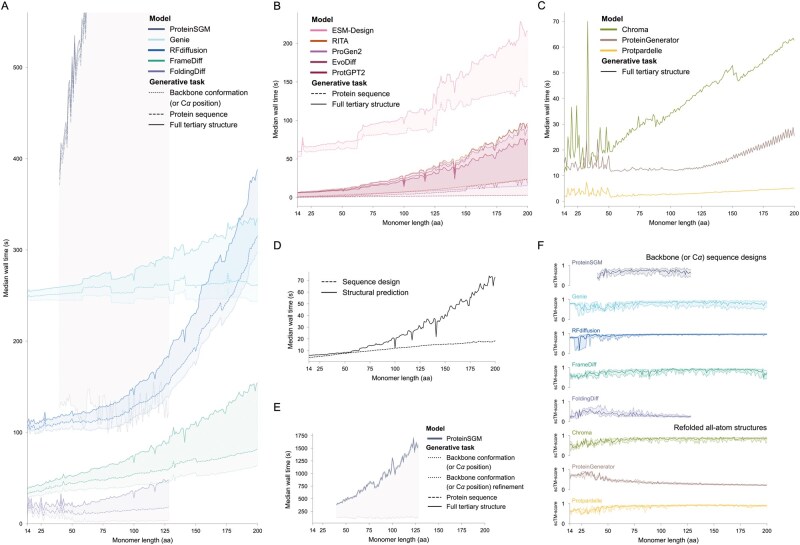
Performance of selected generative models with respect to median generation wall time and designability **A.**–**C.** Median wall times required for unconditional generation of all-atom monomer designs with generative models — models producing backbone conformations (or Cα positions, in the case of Genie) and requiring downstream sequence design and structural prediction (A), models producing protein sequences and requiring downstream structural prediction (B), models producing full tertiary structures with no downstream steps required (C) — are shown as a function of monomer length. The wall times generally increase in an O(n) or O(n^2^) fashion as monomer length increases. **D**. Downstream steps of sequence design and structural prediction, where needed, generally represent a small fraction of the total wall time required to produce an all-atom design. **E**. Expanded view of outlying median wall times per design for ProteinSGM (largely due to the inclusion of a physical backbone refinement step). **F**. Agreement between generated backbone conformations (or Cα positions, in the case of Genie) and putative full tertiary structures, and between generated full tertiary structures and structures predicted by OmegaFold for their primary sequences. An scTM-score approaching 1 indicates strong agreement. Centre lines represent the median value per-length. Outer lines indicate the two inner quartiles. scTM-score, self-consistency TM-score; aa, amino acid.

We generated approximately 3000 unconstrained all-atom protein monomer designs of 14–200 residues in length using each selected generative model (**Figure 1**A–C, Figures S1 and S2; Table S1). Across all models, the observed median wall time required to produce an all-atom design within this length range generally follows an O(n) or O(n^2^) relationship with respect to length. One notable exception is Genie, for which the median generation wall time does not noticeably increase with monomer length. This is likely due to the fact that Genie generates only the C*α* atom positions of the backbone; therefore, the complexity of the task scales more slowly than other structural diffusion models, which must generate positions for many and increasingly more atoms. Generative models of full tertiary structures and sequences appear to require shorter median wall times for inference than those generating backbone conformations. However, the median confidence in the final predicted tertiary structures is consistently higher for generative models of backbone conformations (Figures S3 and S4). While the speed of the structural diffusion models of full tertiary structures may seem counter-intuitive, given that other methods disregard side chains to increase efficiency, Chroma [16] and Protpardelle [17] were both developed with a clear emphasis on optimisation.

The additional wall time required to conduct sequence design (ProteinMPNN [19,20]) and structural prediction (OmegaFold [21]), as required by many models, was not a limiting factor in this length range compared to initial model inference (Figure 1D). The exception is ProteinSGM, which requires an additional, time-intensive step of backbone refinement using Rosetta MinMover [22,23] (Figure 1E). However, this is only the case for a single use of these tools. In reality, most protein design approaches perform multiple iterations of sequence design or structural prediction before selecting the best candidate. Conversely, while a single ProteinSGM generation is significantly slowed by the use of Rosetta MinMover, this can be offset over multiple generations through CPU batch processing.

The designability of generated backbone conformations was assessed by the agreement between the backbone conformation and the final full tertiary structure. We generated 10 putative sequences for each backbone output, and calculated the self-consistency TM-score [24] (scTM-score) between their predicted structures and the generated backbone (Figure 1F). The designability of the backbone conformations appeared to be inversely related to the median generation wall time. RFdiffusion consistently produced final designs with the highest median confidence in the predicted structure and agreement with the generated backbone (Figures S3 and S4), but also exhibited the poorest scaling of generation time with respect to monomer length.

Designability is consistently weaker for shorter monomers, becoming more stable at protein lengths more commonly observed in nature. Similar results were observed with respect to scRMSD (Figure S3). We also calculated scTM-scores between the outputs of our three generative models of full tertiary structures and a structure predicted for each of their primary sequences by OmegaFold (Figure 1F). Chroma and Protpardelle displayed similar performance to the more performant backbone models in this metric, while consistently low agreement between generated and refolded structures was observed for ProteinGenerator [18].

#### Feasibility

**Figure 2:**
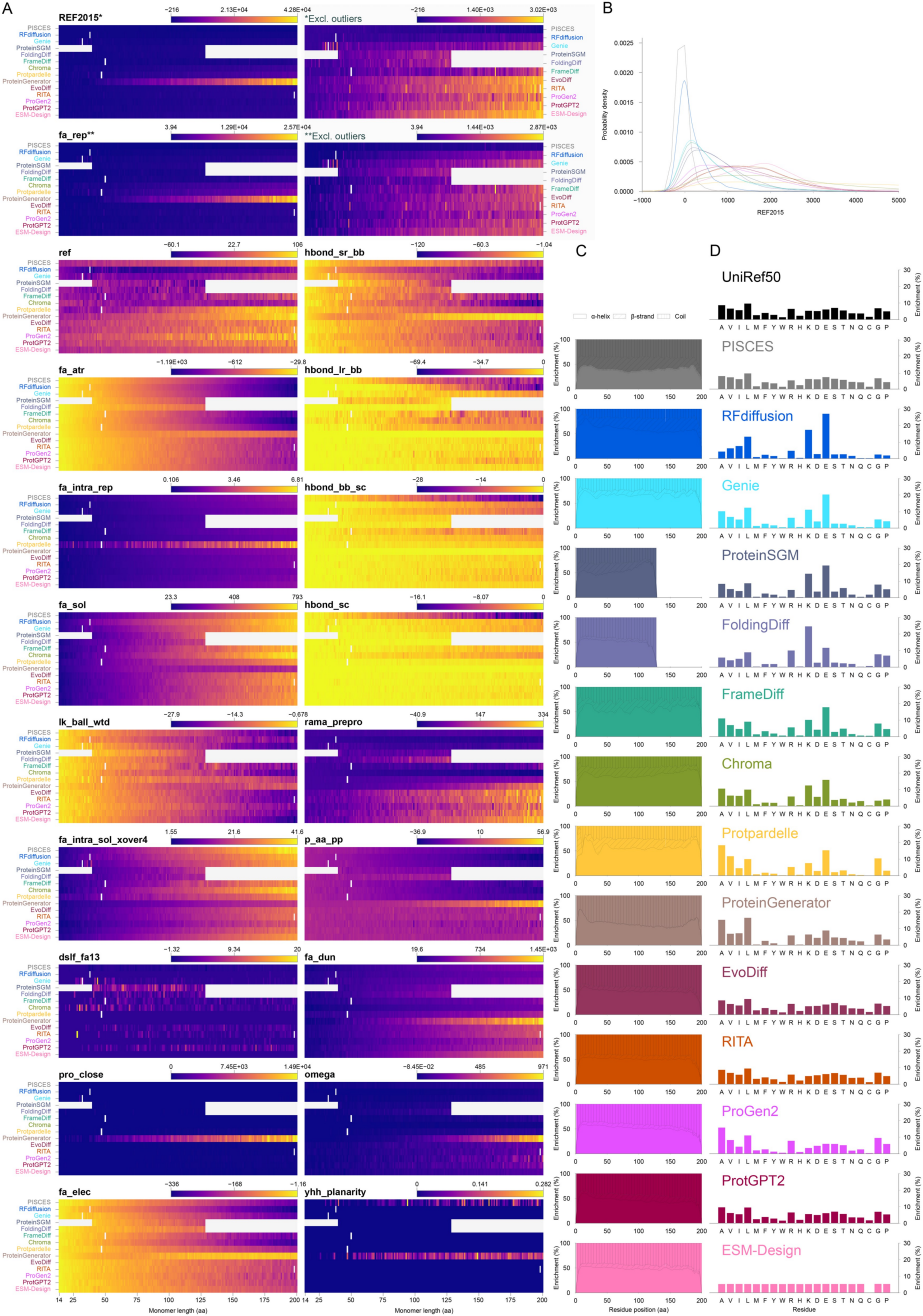
Biochemical profile of generative AI proteins **A**. REF2015 score and subterm values for our generated monomers and a subset of resolved protein chains from the PDB (PISCES), plotted against monomer length. Plots for REF2015 (*) and fa_rep (**) were also generated, with large outlier values observed for monomers generated by ProteinGenerator, Protpardelle, and Chroma removed. **B**. Probability density of REF2015 values for our generated monomers and PISCES chains estimated using KDE. C. Enrichment for α-helix, β-strand, and coil secondary structures per residue position for our generated monomers and PISCES chains. **D**. Overall enrichment of each amino acid in our generated monomers, PISCES chains, and the UniRef50 dataset. REF2015, Rosetta Energy Function 2015; PDB, Protein Data Bank; KDE, Kernel Density Estimation.

We surveyed the sequence content and the geometric and energetic qualities of our generated monomers, compared to a subset of experimentally resolved protein chains from the Protein Data Bank (PDB) obtained via PISCES [25], using PyRosetta [26] (**Figure 2**, Figures S5–S10). Both the experimentally resolved chains and our generated designs exhibited largely uniform amino acid preferences with respect to residue position (Figure S7), but diverging patterns were observed in the overall enrichment for different amino acids throughout their sequences. EvoDiff [6] and the three autoregressive language models appeared to best capture the natural distribution of amino acid preferences, while ESM-Design monomers exhibited an unusual uniform preference for all residues except cysteine. A consistent enrichment in lysine and glutamic acid, as well as a depletion of asparagine, glutamine, histidine, tryptophan, and serine, was observed in monomers generated by structural diffusion models.

β-strands were often under-represented in the secondary structures of generated designs compared to the experimentally resolved chains, particularly those from generative models of sequences. RFdiffusion, FrameDiff [15], Chroma, and Protpardelle appeared to best capture the natural distribution of secondary structure motifs with respect to residue position. With the exception of ProteinGenerator, all models were able to recreate the distributions of Φ, Ψ, and ω angles observed in experimentally resolved chains (Figure S8). All models generated structures with narrower distributions of N-C*α*, C*α*-C, and C-O bond lengths than those observed in the experimentally resolved chains, with the exception of Protpardelle, where the distributions were far broader than those observed for naturally occurring proteins. With the exception of Chroma, all models generated structures with a broader range of C-N bond lengths than those observed in the experimentally resolved chains (Figure S9). Designs from ProteinGenerator often exhibited irregular χ angle distributions in their side chains (Figure S10).

With the exception of RFdiffusion, all models generated monomers with much higher total Rosetta energies (Rosetta Energy Function 2015, REF2015) [27,28] than those observed in the experimentally resolved chains. This is largely a consequence of high Lennard-Jones repulsion values between atoms in different residues (fa_rep). Designs from ProteinGenerator and other generative models of full tertiary structures exhibited particularly high values for fa_rep; however, this was less prominent in the structures predicted for their primary sequences by OmegaFold (Figure S6).

The distributions in the energetic sub-terms of REF2015 varied between different generative models, as well as between generated designs and experimentally resolved chains. Consistent with our observations in overall amino-acid enrichment, designs generated by structural diffusion models differed from the experimentally resolved chains in reference energies for each amino acid (ref). Protpardelle designs exhibited higher Lennard-Jones repulsion between atoms in the same residue (fa_intra_rep) than other models and experimentally resolved chains. With the exception of FoldingDiff and, in some cases, Protpardelle, structural diffusion models consistently differed from sequence-based models in Lennard-Jones attraction between atoms in different residues (fa_atr), inter-residue Lazaridis-Karplus solvation energies (fa_sol), coulombic electrostatic potentials (fa_elec), short-range backbone hydrogen bond energies (hbond_sr_bb), backbone torsion preferences (rama_prepro), penalties for deviant dihedral angles (omega), and backbone-dependent probabilities of amino acid identity (p_aa_pp). Structural diffusion appeared to give more realistic results for fa_atr, fa_elec, rama_prepro, omega, and p_aa_pp, while sequence-based models gave more biologically plausible values for fa_sol and hbond_sr_bb. Nearly all models struggled to recreate reference distributions for sidechain–backbone and sidechain–sidechain hydrogen bonding energies (hbond_bb_sc and hbond_sc), sidechain hydroxyl group torsion preferences (yhh_planarity), disulfide bridge energies (dslf_fa13), and long-range backbone hydrogen bond energies (hbond_lr_bb).

#### Diversity and novelty

**Figure 3:**
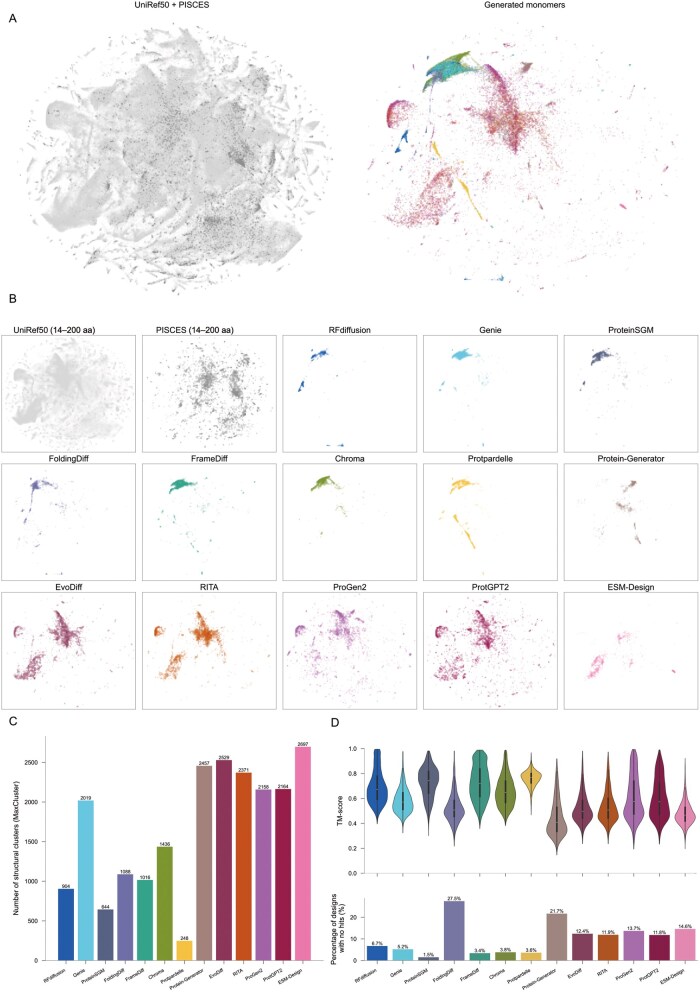
Diversity of naturally occurring and generative AI proteins **A**. *t*-SNE visualisations of the “protein universe” embedded in the ESM large language model, comprising the sequences of a 1% subsample of UniRef50, our experimentally resolved protein chains (PISCES), and our generated monomers. B. *t*-SNE visualisations of sequences (14–200 aa), shown separately by source. **C**. Number of structural clusters detected in the pool of generated monomers (14–200 aa) for each model. **D**. Top: TM-score distribution for the best hits for our generated monomers when queried against the ESMAtlas30 database using Foldseek. Bottom: percentage of generated designs with no hits. *t*-SNE, *t*-Distributed Stochastic Neighbor Embedding.

To explore the distribution of generated monomers throughout the protein universe, we embedded their sequences, the sequences of the PISCES chains, and a 1% subsample of the UniRef50 dataset [29] in the ESM large language model [30], and performed *t*-Distributed Stochastic Neighbor Embedding (*t*-SNE) dimensionality reduction [31,32] on the embedding vector to two dimensions (**Figure 3**). Chains from the PISCES set populated, in a length-dependent manner, a similar overall region of this two-dimensional (2D) space to that of the UniRef50 sequences, although some specific high-density clusters were less apparent (Figure S11). We also assessed the structural diversity of our generated designs by quantifying the number of structural clusters detected by MaxCluster (http://www.sbg.bio.ic.ac.uk/∼maxcluster/; accessed October 18, 2024), and their novelty, by querying each against the ESMAtlas30 database [30] using Foldseek [33]. The uniform distribution of cluster labels and TM-scores observed in our projections (Figures S12 and S13) suggests that distances in this space are likely more indicative of sequence differences than structural ones.

Similar patterns in distribution throughout this space can be observed between generative models of the same paradigm. Monomers generated through structural diffusion appeared to only occupy a small region in comparison to both the UniRef50 and PISCES sequences, whereas generative models of sequences appeared to more evenly populate the space of natural proteins of similar length. The dispersion of monomers from ProteinGenerator, and ESM-Design lay somewhere between the two. Monomers generated by RFdiffusion were the most highly localised within this space, while those from ProtGPT2 had a distribution most similar to that seen in the UniRef50 dataset. Designs from sequence-based models also displayed a larger total number of structural clusters than structural diffusion methods, with the exception of Genie, whilst designs from Protpardelle exhibited the least structural variety. RFdiffusion and FrameDiff produced a more even distribution of cluster sizes compared to other structural diffusion models (Figure S14). Generative models of sequences also had a higher tendency towards structural novelty in their designs, with lower TM-scores for their closest Foldseek query hits and a higher frequency of queries with zero hits. The exception was FoldingDiff, which displayed a similar level of structural novelty in its designs to that of generative models of sequences and the highest incidence of queries with zero hits. Similar results were observed when considering all hits (Figure S15).

### Conditional design around a set of functional motifs

#### Conditional generation of TEV protease designs

RFdiffusion, Chroma, Protpardelle, ProteinGenerator, and EvoDiff were selected to redesign the TEV protease by performing conditional generation of a 237-residue monomer around 5 fixed motif regions. Genie, FoldingDiff, FrameDiff, and ESM-Design do not currently support conditional generation, while ProteinSGM cannot generate monomers of sufficient length. Autoregressive protein language models are only capable of conditional design around a fixed motif at the beginning of a sequence.

Conditional designs by ProteinGenerator were produced using two approaches: specifying only the motif sequences, and specifying the full structural motifs. Similarly, Chroma and RFdiffusion designs were produced by specifying the structural motifs from both the TEV protease monomer in isolation and in complex with its recognition sequence. Additionally, sequence redesign of the backbones generated by RFdiffusion was conducted with both full flexibility and motif sequences fixed. As a comparison, we also generated TEV-based protease designs using only ProteinMPNN sequence design with fixed motif sequences. One hundred TEV protease designs were generated for each approach (Table S2), and the ten sequences for each model with the lowest root mean square deviation (RMSD) relative to template structures in the motif regions were selected for synthesis and *in vitro* validation.

#### Functional validation

**Figure 4:**
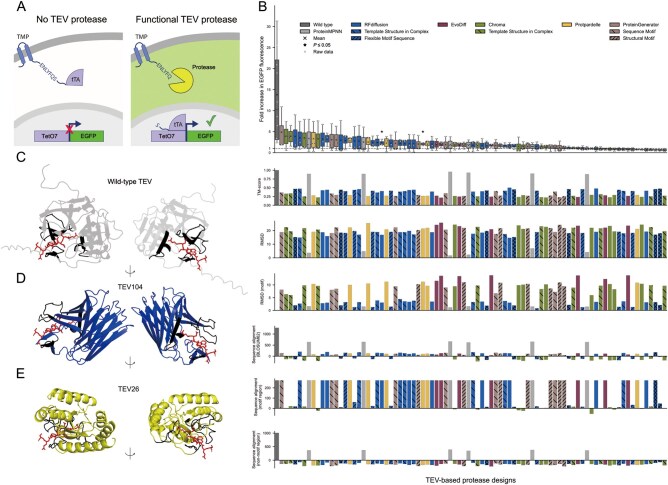
*In vitro* validation of conditional designs around a set of functional motifs from the TEV protease **A**. Schematic diagram illustrating tTA localisation within the cell in the absence and presence of a functional TEV protease. Upon protease cleavage, tTA translocates to the nucleus and induces EGFP gene expression. **B**. Eighty-seven TEV designs generated using 13 different models were ranked by their enzyme activity, measured as the fold increase in EGFP fluorescence relative to the negative (no TEV) control (mean across 3 technical replicates). No clear trend was observed between enzyme activity and the pairwise structural and sequence alignment scores (TM-Score, RMSD, and BLOSUM62) of the designs against the template structure. **C**. Template structure of wild-type TEV protease S219V (grey) used for conditional designs in complex with the recognition sequence (red). **D**. TEV104, a TEV protease design produced by RFdiffusion. **E**. TEV26, a TEV protease design produced by Protpardelle. In (C–E), functional motifs are highlighted in black. TEV, tobacco etch virus; TMP, transmembrane protein; tTA, tetracycline-controlled transactivator; EGFP, enhanced green fluorescent protein; TetO7, seven-repeat Tet operator; RMSD, root mean square deviation.

To compare the catalytic activity, designed monomers were expressed in baby hamster kidney-21 (BHK-21) cells together with a tetracycline-inducible enhanced green fluorescent protein (EGFP) and a synthetic protein consisting of tetracycline-controlled transactivator (tTA) tethered via a linker containing the TEV cleavage site (ENLYFQ↓S) to a transmembrane protein (TMP). The TMP-fused tTA is localised to the plasma membrane, and thus the EGFP signal is low in the absence of an active TEV protease. However, an active protease cleaves tTA, enabling its translocation to the nucleus and induction of EGFP expression (**Figure 4**A).

Of the 110 selected design monomers, 23 could not be successfully cloned, potentially due to the instability or toxicity of the synthesised sequence towards the host *Escherichia coli* cells. We evaluated the functional activity of 87 TEV designs and ranked them based on their enzyme activity, measured as a fold increase in EGFP fluorescence compared to the negative (no TEV) control (Figure 4B). Among these, 34 designs exhibited a greater than 2-fold increase in EGFP fluorescence, suggesting potential protease activity; however, only two designs (TEV104 and TEV26, generated using RFdiffusion and Protpardelle, respectively) were found to have nominally significant activity (*P* = 0.045 and *P* = 0.023, respectively) compared to the negative control, though this was not significant following Bonferroni correction. The contact residues within the motif of the wild-type TEV protease (Figure 4C) are conserved and in close contact with the target substrate sequence in both TEV104 and TEV26 designs, suggesting critical interactions are preserved (Figure 4D and E). Additionally, these residues demonstrate a similar spatial orientation around the target substrate, indicating recapitulation of key interaction motifs necessary for substrate binding and cleavage activity. Interestingly, there was no discernible relationship between the design strategy and protease activity in the tested TEV designs, and the efficiency was still far less compared to the wild-type protease, suggesting the need for further optimisation.

## Discussion

We adopted a rational, quantitative approach to systematically benchmark generative protein models. In initial reports [6–18], the qualitative performance of these models has often been assumed from the degree of model fit to training data, or evaluated from a few selected outputs. The lack of a systematic approach has resulted in an incomplete understanding of the merits and shortcomings of current popular approaches, the rationale for their use, or potential complementary strategies. The trends we have observed across our selected metrics for monomer designability, feasibility, diversity, and novelty have highlighted performance trade-offs between models and revealed fundamental differences between the two popular paradigms of structural diffusion and protein language modelling. Additionally, we have assessed certain practical considerations of running these models, with the goal of facilitating functional and accessible *de novo* design.

Models based on structural diffusion generally produce designs with higher confidence in the putative structure, and more biochemically appropriate distributions of energy terms and secondary structure features. However, they are less structurally diverse, and exhibit a strong bias towards particular sequence content that is inconsistent with both data comparable to their training examples (PISCES chains) and the larger space of known protein sequences (UniRef50). While it is possible that this bias towards particular sequence content and limited structures is introduced during sequence design with ProteinMPNN, these results are also observed for Chroma, which uses its own novel sequence design network.

Structural diffusion models appear to have learned to generate a narrow range of outputs, rather than effectively capturing or generalising beyond the distribution of their training data. The inclusion of side-chain influences in the generation process does not appear to improve this limited diversity. Interestingly, the tertiary structures produced by such methods are also less biochemically feasible than the structures predicted for their primary sequences by OmegaFold, largely due to poor side-chain conformations. However, considering the short generation times achieved by these methods, incorporating a post-generation “refolding” step is a feasible strategy to overcome this.

Protein language models generally produce a diverse range of designs that are more likely to be novel, but typically have lower confidences in their putative structures, and are potentially less biochemically plausible. Nevertheless, it is less clear whether this represents a higher level of intrinsic disorder for these sequences, or reflects the limitations of the structural prediction method over the more diverse space of sequences explored by these models. The sequence diffusion approach of EvoDiff appears to generally result in similar designs to the autoregressive language models. ProteinGenerator, a model that combines sequence diffusion with a structure-aware objective function, largely produces biochemically unrealistic tertiary structures. ESM-Design also exhibits relatively poor performance, with a near-uniform residue preference observed in its generated sequences. Nevertheless, given the significant reduction in iterations necessary for inclusion, this observation likely reflects the computational power required to use this tool effectively, rather than its inherent capabilities. Indeed, when the number of iterations used by ESM-Design was increased to 10,000 — requiring approximately 4 h to generate a single 100-residue sequence on Colab — the resulting sequences showed a marked departure from this uniform residue preference (Figure S16; see Materials and methods). Similarly, our decision to replace the default Rosetta FastDesign and FastRelax steps with ProteinMPNN and OmegaFold as the all-atom design protocol for ProteinSGM may have impacted its performance. However, we observed that while ProteinSGM backbones subjected to side-chain minimization with FastDesign remained relatively consistent with the original backbone, the final all-atom refined models from FastRelax showed substantial divergence from the initial backbones. This suggests that this physical design approach compromises the generative model’s designability (Figures S17 and S18; see Materials and methods).

For the five models capable of executing our TEV protease redesign task, we found no significant evidence of increased activity over the negative control or meaningful differentiation between different models or design strategies upon functional validation. This suggests that further improvements are needed before these models, all based on sampling from learned diffusion processes, can complete such tasks without substantial oversight and optimisation. More iterative protein design approaches, such as SCUBA [34], which continuously samples and optimises backbone structures using a learned energy function, may be better suited to such tasks.

Even when data distributions are explicitly modelled, and likelihoods are tractably computed, these factors alone do not ensure high-quality, diverse proteins or generalisation beyond current training datasets. Many current models generate incomplete representations of proteins, such as sequences or backbone conformations, making the quality and diversity of their designs dependent on the predictive models used alongside them. By directly examining the attributes of their outputs, we have systematically compared these models at the point most relevant to *de novo* design.

While our benchmarking focused on monomeric proteins, we acknowledge the growing focus on multichain systems such as enzymes, antibodies, and protein complexes [35]. Extending generative models to multichain design presents key challenges, such as accurately capturing inter-chain structural coupling, maintaining interface specificity, and scaling to the increased sequence and conformational complexity. Nevertheless, monomer-focused models provide a critical foundation by enabling the controlled generation of well-folded scaffolds. Future developments in generative AI may involve integrating explicit interface modelling, co-folding prediction (*e.g.*, through AlphaFold-Multimer or similar tools [5]), and conditioning generation on inter-chain contact maps or docking constraints. Overcoming these challenges will be essential for transitioning from monomeric design to functional, multimeric assemblies with real-world therapeutic and industrial utility.

## Materials and methods

### Generation of protein designs

All protein generations, backbone sequence designs, and structural predictions were performed using a T4 GPU on the Google Colab platform. Sequence designs were conducted on C*α*-only backbone structures using ProteinMPNN [19,20]. Ten prospective sequences were generated per backbone at a sampling temperature of 0.1. Structural predictions were conducted with OmegaFold [21] using default parameters.

### Unconditioned monomer designs

For each of the 13 generative models, a distribution of lengths between 14 and 200 residues (Figure S2**)** was sampled using an empirical cumulative distribution function estimated from the repartition of Swiss-Prot sequences by size reported by the UniProt [29] statistics portal (accessed January 2024). Unconditioned monomer designs were generated at these lengths using the default parameters for each model as described in their documentation unless otherwise noted. Designs at each length were batched where possible, with generation wall time assumed to be evenly distributed between designs. For autoregressive large language models, where only a maximum length can be specified at inference, random-length monomers were generated with an appropriate maximum-length parameter until the desired distribution could be subsampled.

Existing Google Colab implementations were available for RFdiffusion and Chroma and were used without alteration beyond the recording of metadata. Minimal adjustments were made in the implementation of other models to enable execution on Google Colab, facilitate batching, manage GPU memory, and reduce model reloading between generations. Generations with ESM-Design and Genie were unable to be batched due to high GPU memory usage. The Rosetta refinement step for ProteinSGM was conducted on a Google Colab CPU instance, as suggested by its documentation [13].

The default Rosetta FastDesign and FastRelax steps were excluded from ProteinSGM generations due to long compute times, and ESM-Design was run with a significantly reduced number of iterations (250, down from the default 170,000) to make its generation wall time comparable to that of other models. To quantify the effect of these parameter changes, a set of 30 100-residue sequences was generated with ESM-Design using 10,000 iterations, and the 17 refined 100-residue backbones generated by ProteinSGM (with Rosetta MinMover) were reprocessed into full atomic structures with the ProteinSGM FastDesign and FastRelax steps enabled.

### Conditional design around defined TEV protease motifs

#### Definition of functional motifs and creation of template structures

A structure was predicted for the TEV protease S219V sequence (reported by Packer et al. [36]) in complex with its recognition sequence using AlphaFold [3] as implemented in ColabFold [37]. This structure was then used to create template structures. Interface residues between the two chains were identified in PyMol (www.pymol.org/; accessed October 18, 2024) using a script by Jason Vertrees (https://pymolwiki.org/index.php/InterfaceResidues; accessed October 16, 2024), and used to define five fixed motif regions to supply to the generative models (residues: 28–33, 47–51, 140–152, 168–179, 212–221).

#### Generation of conditioned monomers and complexes

Motif regions were provided to each model for conditional generation as outlined in their documentation. Sequence design was conducted on the RFdiffusion backbones with ProteinMPNN defaults and with an overwhelming bias provided towards the motif residues in the motif region. Ten putative sequences were designed for each backbone, and the sequence with the structure most consistent with the backbone (highest scTM-score [24]) was selected. The biased ProteinMPNN approach was also applied directly to the template structure as a comparison.

#### Selection of designs

Ten TEV scaffold designs were selected for each conditional design approach. Designs were filtered to retain those in the bottom quartile of REF2015 scores [27,28]. Subsequently, the ten designs with the lowest RMSD relative to template structures in the motif regions were selected (Table S2). The tertiary structures of Chroma, Protpardelle, and ProteinGenerator designs were refolded with OmegaFold [21] before selection, due to the high REF2015 scores observed for their initial outputs. A list of repositories for each model used in this study can be found in Table S3.

### *In silico* analysis

All computational analyses of designs were performed on the Tasmanian Partnership for Advanced Computing (TPAC) Rosalind HPC cluster using multiple Intel(R) Xeon(R) CPU E5-2680 v4 processors.

The PISCES server [25] was used to create a comparison dataset of protein chains from the PDB fulfilling the following criteria: determined by X-ray diffraction, maximum pairwise sequence identity = 25, minimum resolution = 0.0, maximum resolution = 2.0, maximum R-value (X-ray) = 0.25, minimum chain length = 40, maximum chain length = 10,000, including chains with breaks and missing residues due to disorder. The .pdb files were cleaned using pyrosetta.toolbox.cleaning.cleanATOM() [26].

Outputs from Chroma in .CIF format were converted to .pdb format using PyMOL.

#### Designability

Folded structures from backbone sequence designs and refolds of full tertiary structure outputs were aligned to their respective backbones or original designs. The scTM-score [24] and scRMSD were calculated using the tmtools Python package and Biopython’s SVDSuperimposer module, respectively. The backbone sequence design with the highest scTM-score was chosen to represent the design in all further analyses (Table S1). scRMSD calculations between all-atom designs and refolds were performed on an all-atom basis.

#### 2D visualisation

The mean embeddings over the sequences of all unconditioned monomer designs, PISCES chains, and a 1% subsample of the UniRef50 dataset were extracted from the final layer of the ESM-2 language model [30] (esm2_t33_650M_UR50D) using the esm-extract command-line interface. These 280-dimensional representations were then reduced to 2D coordinates for visualisation using *t*-SNE-CUDA [31,32].

#### Novelty

Unconditioned monomer designs were queried against the ESMAtlas30 dataset [30] to retrieve TM-score normalised by query/target length, average alignment local distance difference test (LDDT), and homology probability using Foldseek [33].

#### Diversity

For each generative model, structural clusters were assigned to unconditioned monomers at each length with MaxCluster (http://www.sbg.bio.ic.ac.uk/∼maxcluster/; accessed October 18, 2024) using sequence-independent, average-linkage clustering: maxcluster64bit -l <pdb_list > -C 2 -in -R1 ./tm_results.txt -Tm 0.5.

#### Feasibility

Phi (Φ), Psi (Ψ), Omega (Ω), and Chi (χ) angles; backbone bond lengths; sequence; secondary structure annotation; and Rosetta REF2015 energy terms [27,28] were calculated for each final monomer design using PyRosetta [26].

### *In vitro* analysis

#### Functional assessment of monomeric TEV designs

##### DNA cloning

A stable and efficient TEV mutant (S219V) [36,38] and synthetic DNA sequences encoding the TEV designs were codon-optimised for expression in BHK-21 cells and purchased as eBlock gene fragments from Integrated DNA Technologies (Coralville, IA). The gene fragments were cloned into a mammalian expression vector (pCMV-phenomycin) using NEBuilder HiFi DNA Assembly Master Mix (Catalog No. E2621L, New England Biolabs, Ipswich, MA). The assembled plasmids were transformed into NEB Stable Competent *E. coli* grown in Luria-Bertani broth or agar media supplemented with 100 μg/ml carbenicillin. Plasmids were purified using the Monarch Spin Plasmid Miniprep Kit (Catalog No. T1110, New England Biolabs).

##### *In vitro* TEV activity assay

A tTA-based fluorescent reporter system was used to determine the protease activity of the TEV designs [38]. The reporter system comprised two components: (1) a tTA fused to a TMP via a peptide linker containing the TEV cleavage site (ENLYFQ↓S); and (2) an EGFP expressed under the control of a CMV promoter (the CMV-RHO2-TEVcs-tTA plasmid) and a seven-repeat Tet operator (the TetO7-EGFP plasmid), respectively.

The reporter and TEV design plasmids were co-transfected into BHK-21 cells using TransIT-X2 (Catalog No. MIR6005, Mirus Bio LLC, Madison, WI) following the manufacturer’s protocol. Cells were seeded onto 48-well cell culture plates at a density of 2.5 × 10^4^ cells per well one day prior to transfection. A transfection complex was prepared containing 100 ng of each of the following plasmids: CMV-TEV design, CMV-RHO-TEVcs-tTA, and TetO7-EGFP, along with 30 µl Opti-MEM (Catalog No. 31985070, Thermo Fisher Scientific, Waltham, MA) and 0.9 µl TransIT-X2. CMV-TEV designs were randomly assigned in triplicate across 12 plates, with each plate containing a positive (wild-type TEV) and a negative (no TEV) control. The transfection complex was incubated for 20 min at room temperature before being added to the cells. Edge wells contained media only to avoid edge effects. After 24 h, EGFP expression was measured using a Carl Zeiss Axio Vert.A1 Inverted Microscope (ZEISS, Oberkochen, Germany). Images were acquired with a 5× objective lens. The same field of view was used for the corresponding brightfield (BF) image, with auto-exposure settings used for image capture.

#### Image analysis

ilastik [39], a machine-learning tool for bioimage analysis, was used to quantify EGFP expression using its Cell Density Counting module. Within the Cell Density Counting interface, the following “Features” were selected: for EGFP analysis, the Color/Intensity (Gaussian smoothing) with sigma values of 0.30, 0.70, 1.00, 1.60, 3.50, 5.00, and 10.00 for σ0, σ1, σ2, σ3, σ4, σ5, and σ6, respectively; for BF analysis, the Edge (Laplacian of Gaussian) with sigma values of 0.70, 1.00, 1.60, 3.50, and 5.00 for σ1, σ2, σ3, σ4, and σ5, respectively. Under the “Counting” interface, a random forest algorithm was used (Ntrees: 10; MaxDepth: 50), with sigma values of 8 and 2.5 for BF and EGFP analyses, respectively. Separate models were trained for each replicate for EGFP and BF images. Raw images (.jpeg format) were uploaded to ilastik; EGFP-expressing cells were demarcated in the “Foreground”, whereas areas devoid of EGFP signals were defined as “Background”. For cell density counting on BF images, individual foci were indicated as “Foreground” in areas where cell surface area was greatest, whereas “Background” was indicated using a fine “1” size setting for the intervening spaces of cells in images that had adequately sparse density.

To account for the differences in total cell population, the number of EGFP-expressing cells was normalised to the cell count observed in the BF images. EGFP expression in the TEV design group was compared to the negative (no TEV) control group, and the fold increase was calculated by dividing the number of normalised EGFP-positive cells in the TEV design group by that in the negative (no TEV) control group. Statistical analysis was performed using a *t*-test.

## Supplementary Material

qzag014_Supplementary_Data

## Code availability

Code for implementing each model on Google Colab and analysing the generated data is available at https://github.com/hewittlab/Systematic-comparison-of-Generative-AI-Protein-Models.

## CRediT author statement

**Alexander J Barnett:** Conceptualisation, Data curation, Formal analysis, Software, Writing – original draft. **Rajendra KC:** Conceptualisation, Formal analysis, Writing – original draft. **Pratikshya Pandey:** Formal analysis, Writing – review & editing. **Pamodha Somasiri:** Formal analysis, Writing – review & editing. **Kirsten A Fairfax:** Formal analysis, Writing – review & editing. **Sandy Hung:** Formal analysis, Writing – review & editing. **Alex W Hewitt:** Conceptualisation, Writing – review & editing, Project administration, Supervision. All authors have read and approved the final manuscript.
