# Supplementary material for: Benchmarking Generative AI Protein Models Reveals Differences Between Structural and Sequence-based Approaches
Source: Genomics Proteomics Bioinformatics. 2026 Feb 15;24(2):qzag014. doi: 10.1093/gpbjnl/qzag014 (PMC13472679; doi:10.1093/gpbjnl/qzag014)
Supplement: Supplementary file 1 [file qzag014_supplementary_data.zip › Figure S16.pdf]

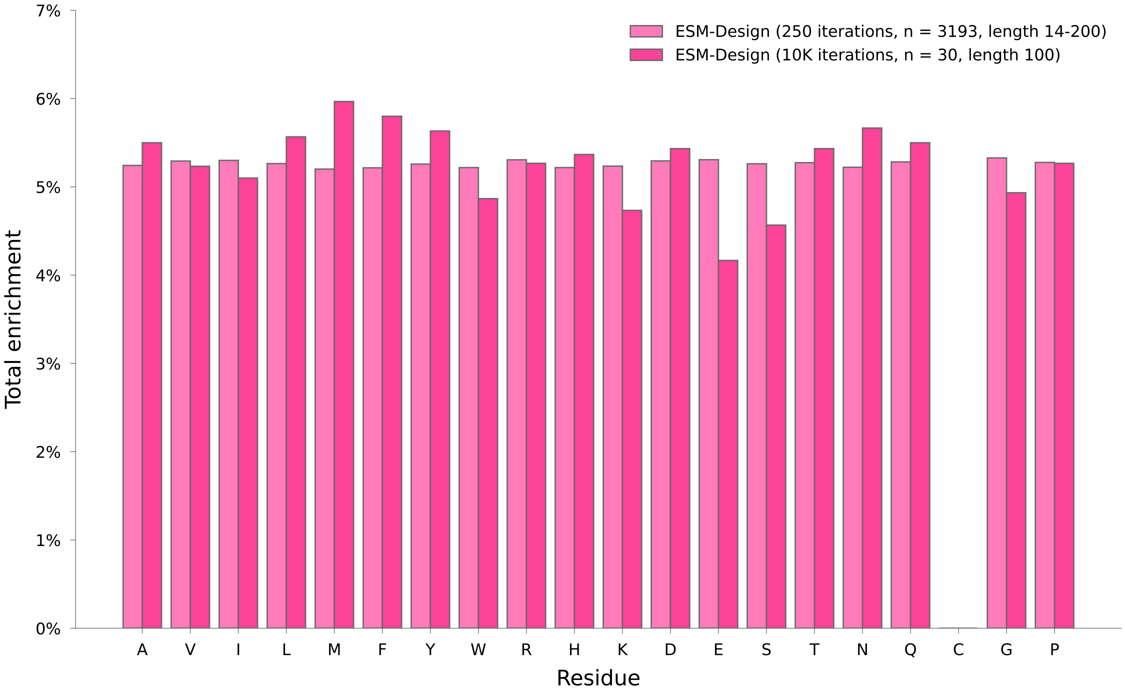

ESM-Design (250 iterations, n = 3193, length 14-200)
ESM-Design (10K iterations, n = 30, length 100)
Total enrichment
7%
6%
5%
4%
3%
2%
1%
0%
A
V
I
L
M
F
Y
W
R
H
K
D
E
S
T
N
Q
C
G
P
Residue
