# Supplementary material for: Benchmarking Generative AI Protein Models Reveals Differences Between Structural and Sequence-based Approaches
Source: Genomics Proteomics Bioinformatics. 2026 Feb 15;24(2):qzag014. doi: 10.1093/gpbjnl/qzag014 (PMC13472679; doi:10.1093/gpbjnl/qzag014)
Supplement: Supplementary file 1 [file qzag014_supplementary_data.zip › Figure S17.pdf]

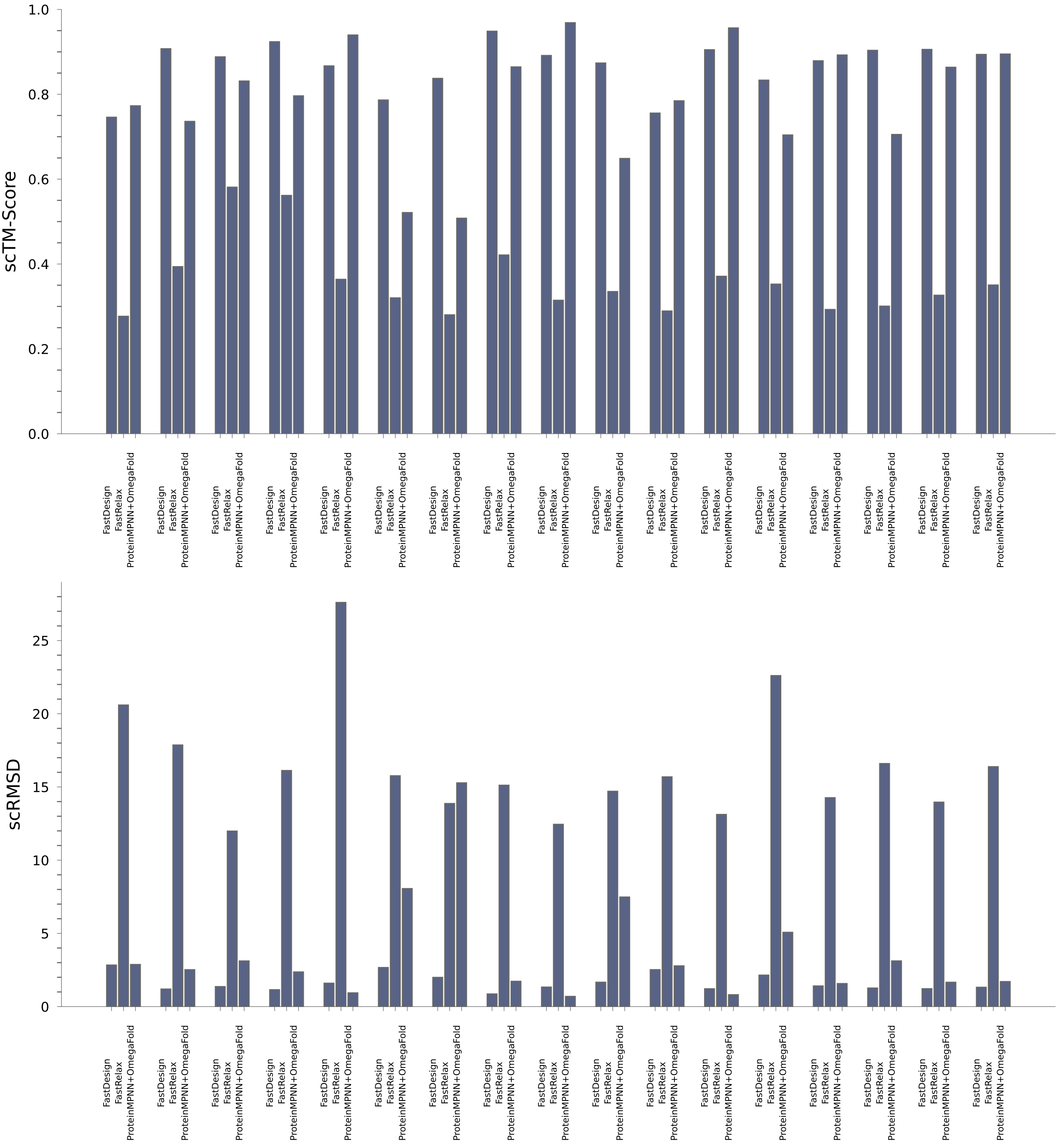
scTM-Score
0.0
0.2
0.4
0.6
0.8
1.0
FastDesign
FastRelax
ProteinMPNN+OmegaFold
scRMSD
0
5
10
15
20
25
