# Supplementary material for: Benchmarking Generative AI Protein Models Reveals Differences Between Structural and Sequence-based Approaches
Source: Genomics Proteomics Bioinformatics. 2026 Feb 15;24(2):qzag014. doi: 10.1093/gpbjnl/qzag014 (PMC13472679; doi:10.1093/gpbjnl/qzag014)
Supplement: Supplementary file 1 [file qzag014_supplementary_data.zip › Figure S18.pdf]

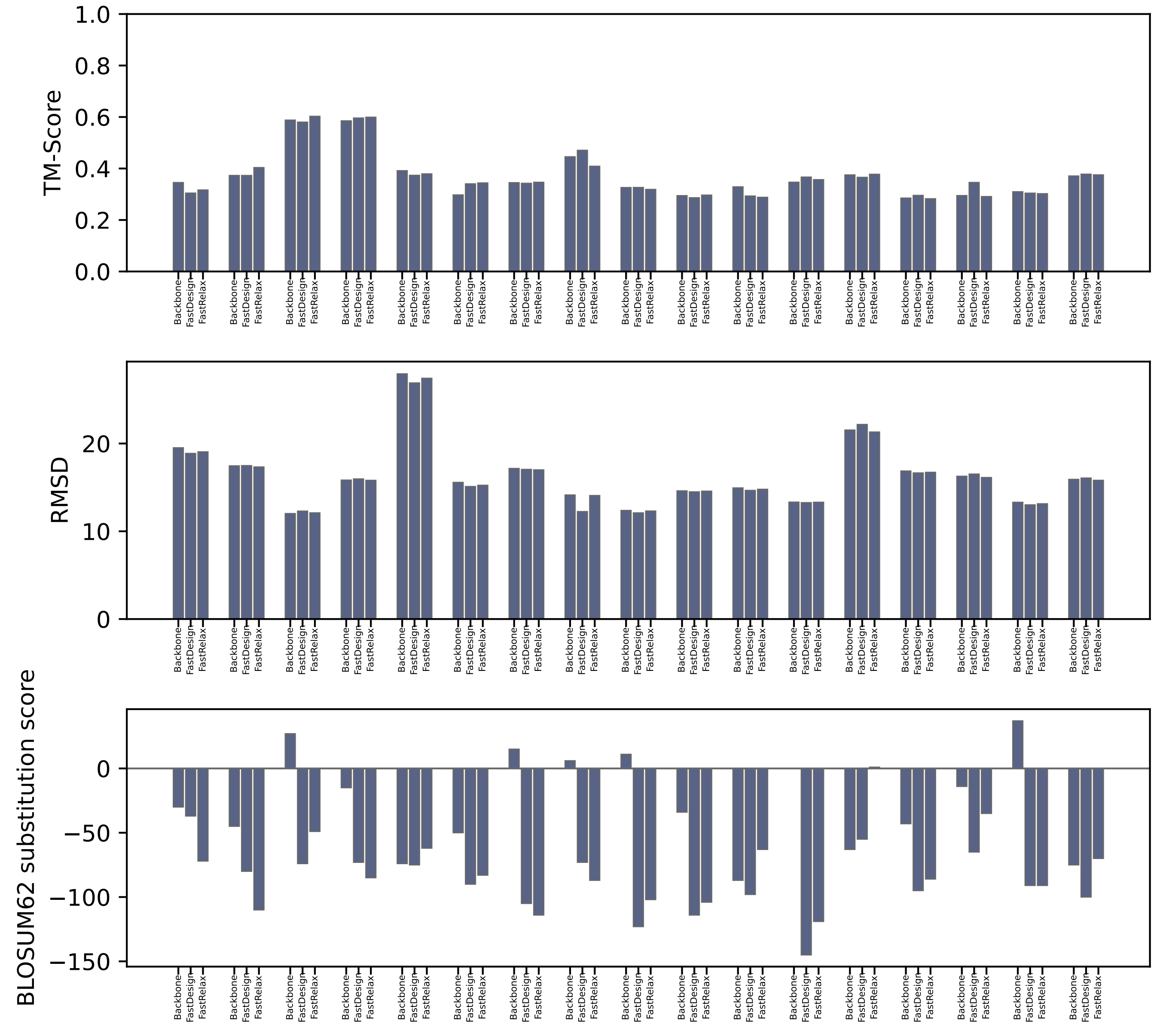

TM-Score
1.0
0.8
0.6
0.4
0.2
0.0
RMSD
20
10
0
BLOSUM62 substitution score
0
−50
−100
−150
Backbone
FastDesign
FastRelax
