# Supplementary material for: Benchmarking Generative AI Protein Models Reveals Differences Between Structural and Sequence-based Approaches
Source: Genomics Proteomics Bioinformatics. 2026 Feb 15;24(2):qzag014. doi: 10.1093/gpbjnl/qzag014 (PMC13472679; doi:10.1093/gpbjnl/qzag014)
Supplement: Supplementary file 1 [file qzag014_supplementary_data.zip › Supplementary material captions.docx]

**Supplementary materials**

**Figure S1 Number of unconstrained all-atom protein monomer designs of between 14 and 200 residues in length generated per each generative model**

FoldingDiff and ProteinSGM were unable to generate monomers outside of their limited output length ranges (≤128 and 40–128 residues, respectively). Number of chains in the PISCES set of experimentally resolved protein chains provided for comparison.

**Figure S2 Distribution of monomer lengths generated with selected models**

Distribution was modelled on the repartition of Swiss-Prot sequences by size as reported by the UniProt statistics portal (Accessed January 2024).

**Figure S3 Agreement between putative tertiary structures/refolds and generated backbones/generated tertiary structures (scTM-Score, scRMSD), and median OmegaFold structure prediction confidence vs generated monomer length**

Centre lines represent the median value per-length. Outer lines define the two inner quartiles. Raw data points are displayed in the model colour, with outliers displayed in black. In the case of Genie, for which only Cα positions are generated, agreement is between the generated backbone Cα atoms and those of the putative tertiary structure.

**Figure S4 Median Omegafold structure prediction confidence for final selected backbone sequence designs and generated sequences vs generated monomer length**

Centre lines represent the median value per-length. Outer lines define the two inner quartiles. Raw data points are displayed in the model colour, with outliers displayed in black.

**Figure S5 Probability density estimation (KDE) of Rosetta Energy Score (REF2015) values and subterms for our generated monomers and PISCES chains**

**Figure S6 Rosetta Energy Scores (REF2015), subterm values, and secondary structure enrichment vs monomer length/position for PISCES chains and the structures predicted by OmegaFold for the primary sequences of monomer designs from Chroma, Protpardelle, and ProteinGenerator**

**A.** Energy Scores and subterm values vs monomer length. **B.** Probability density estimation (KDE) of REF2015 values. **C.** Percent (%) enrichment for alpha helix, beta strand, and coil secondary structures per residue position.

**Figure S7 Percent (%) Amino acid enrichment per position for our generated monomers and PISCES chains**

**Figure S8 Distributions (KDE probability density estimation) of phi (Φ), psi (Ψ), and omega (ω) dihedral angles observed in our generated monomers and PISCES chains**

**Figure S9 Distributions (KDE probability density estimation) of N-Cα, Cα-C, and C-O bond lengths observed in our generated monomers and PISCES chains**

**Figure S10 Distributions (KDE probability density estimation) of side-chain torsion angles ( χ1, χ2, χ3, χ4) observed in our generated monomers and PISCES chains for each residue type (i-XX)**

**Figure S11 Distributions of sequence lengths of a 1% subsample of UniRef50, PISCES chains, and our generated monomers, throughout the t-SNE visualisations of their embedding-vectors in the ESM Large Language Model**

**Figure S12 Distributions of the best-hit TM-Scores for our generated monomers when queried against the ESMAtlas30 database in the t-SNE visualisations of their embedding-vectors in the ESM Large Language Model**

**Figure S13 Distributions of the structural cluster labels identified by MaxCluster for our pools of generated monomers in the t-SNE visualisations of their embedding-vectors in the ESM Large Language Model**

**Figure S14 Distributions of sizes for structural clusters identified by MaxCluster for our pools of generated monomers**

**Figure S15 Distributions of TM-Scores of our generated monomers, by model, when queried against the ESMAtlas30 database**

Swarms represent ~5% of all hits. Larger swarm points represent TM-Scores from best hits.

**Figure S16 Overall enrichment of each amino acid in sequences generated by ESM-Design at 250 iterations and 10,000 iterations**

**Figure S17 Structural Agreement (scTM-Score, scRMSD) between 17 all-atom structures produced by Rosetta FastDesign, Rosetta FastDesign+FastRelax, and ProteinMPNN+OmegaFold and their initial length-100 backbones generated by ProteinSGM**

**Figure S18 Structural (TM-Score, RMSD) and Sequence (BLOSUM62 Substitution) Alignment Scores between 17 all-atom structures produced by Rosetta FastDesign, Rosetta FastDesign+FastRelax, from length-100 backbones generated by ProteinSGM, and their matched all-atoms designs generated using ProteinMPNN+OmegaFold**

**Table S1 Protein monomer designs generated between 14 and 200 residues in length using each selected generative model**

**Table S2 Redesign of the Tobacco Etch Virus (TEV) protease by performing conditional generation of a 237-residue monomer around fixed motif regions**

**Table S3 Repository of models used for benchmarking**
