# Supplementary material for: Benchmarking Generative AI Protein Models Reveals Differences Between Structural and Sequence-based Approaches
Source: Genomics Proteomics Bioinformatics. 2026 Feb 15;24(2):qzag014. doi: 10.1093/gpbjnl/qzag014 (PMC13472679; doi:10.1093/gpbjnl/qzag014)
Supplement: Supplementary file 1 [file qzag014_supplementary_data.zip › Figure S3.pdf]

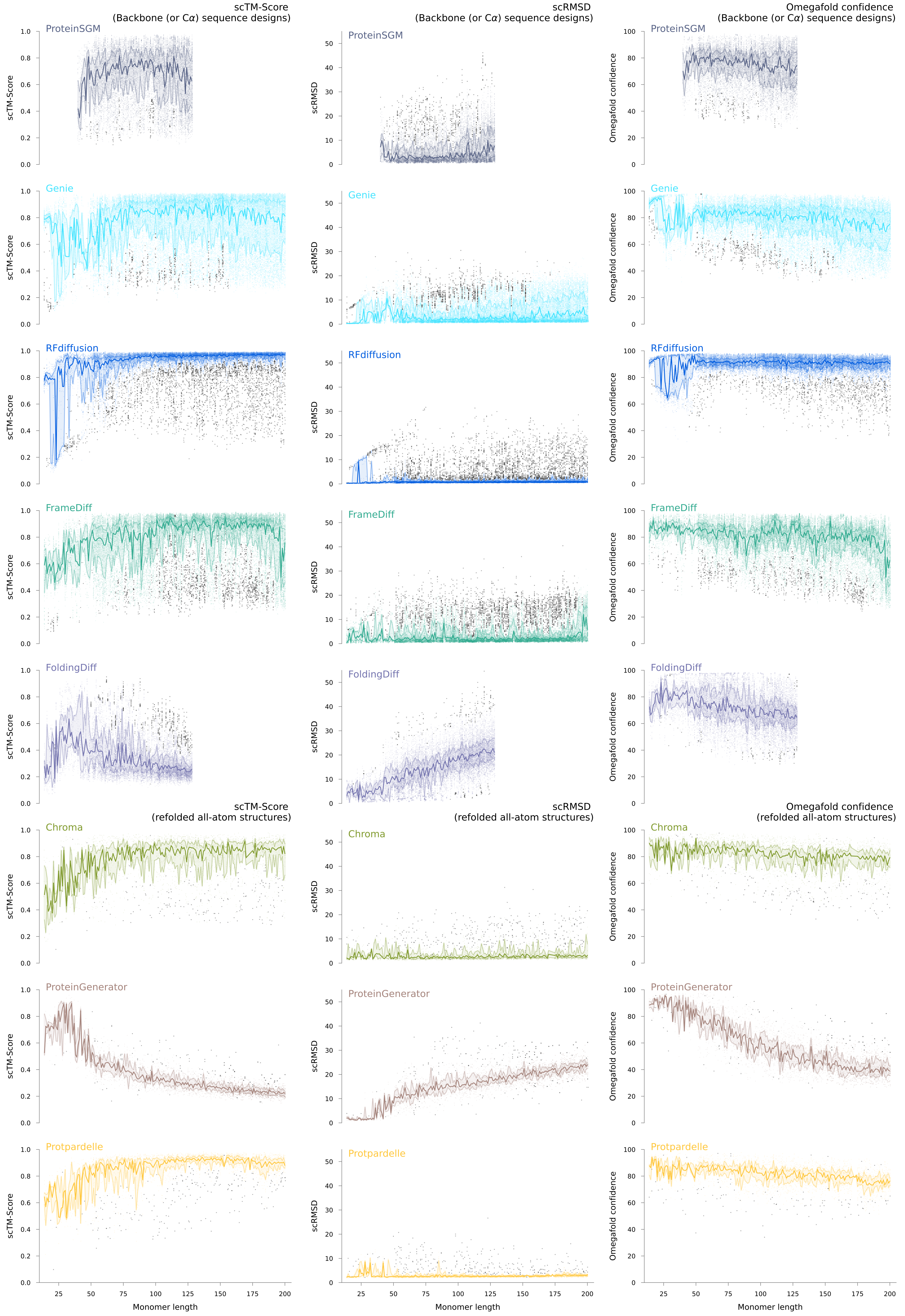

scTM-Score (Backbone (or Cα) sequence designs)
scRMSD (Backbone (or Cα) sequence designs)
Omegafold confidence (Backbone (or Cα) sequence designs)
ProteinSGM
Genie
RFdiffusion
FrameDiff
FoldingDiff
scTM-Score (refolded all-atom structures)
scRMSD (refolded all-atom structures)
Omegafold confidence (refolded all-atom structures)
Chroma
ProteinGenerator
Protpardelle
scTM-Score
scRMSD
Omegafold confidence
Monomer length
