# Supplementary material for: Benchmarking Generative AI Protein Models Reveals Differences Between Structural and Sequence-based Approaches
Source: Genomics Proteomics Bioinformatics. 2026 Feb 15;24(2):qzag014. doi: 10.1093/gpbjnl/qzag014 (PMC13472679; doi:10.1093/gpbjnl/qzag014)
Supplement: Supplementary file 1 [file qzag014_supplementary_data.zip › Figure S4.pdf]

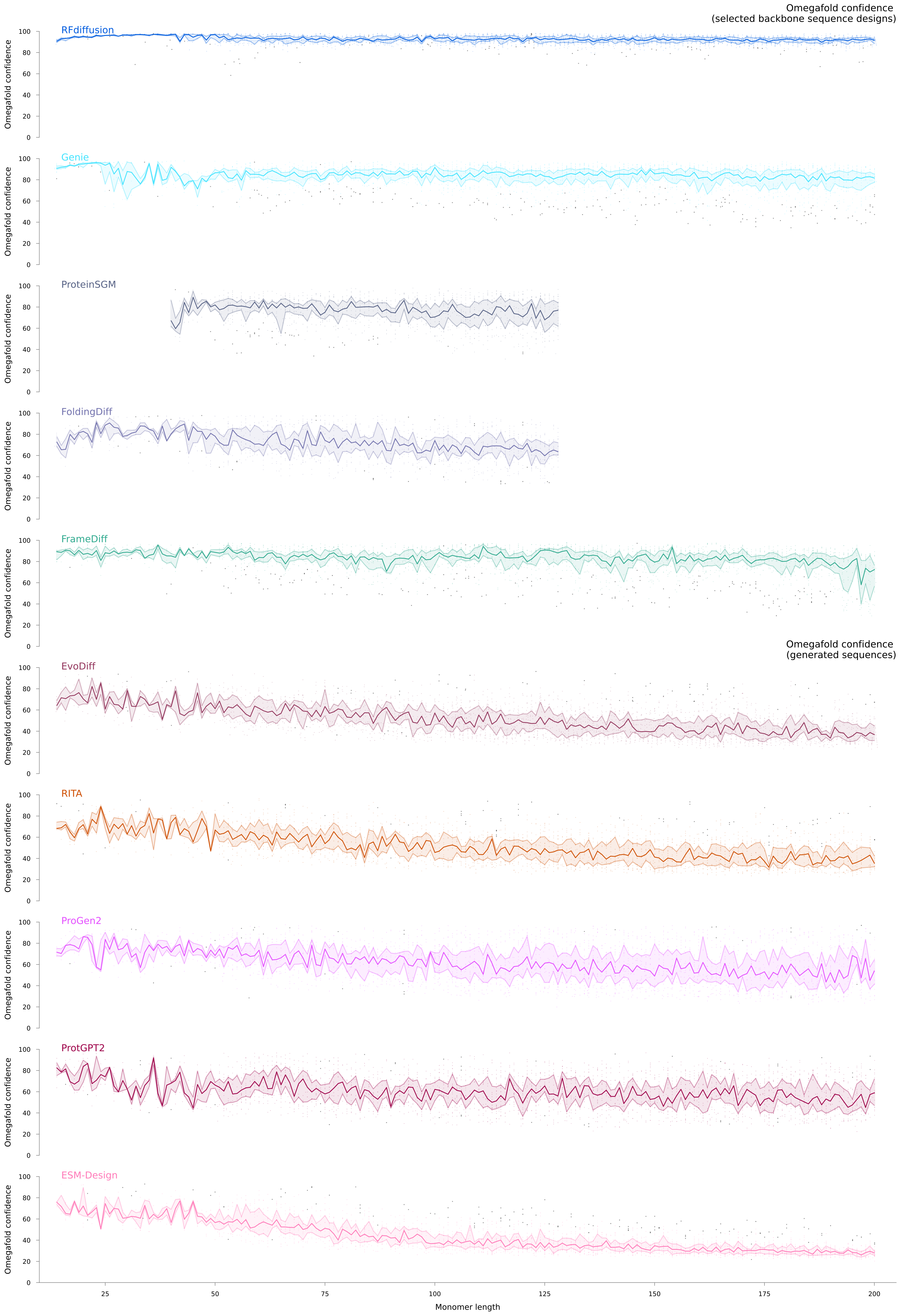
Omegafold confidence
(selected backbone sequence designs)
RFdiffusion
Genie
ProteinSGM
FoldingDiff
FrameDiff
Omegafold confidence
(generated sequences)
EvoDiff
RITA
ProGen2
ProtGPT2
ESM-Design
Omegafold confidence
Monomer length
25
50
75
100
125
150
175
200
