# Supplementary material for: Benchmarking Generative AI Protein Models Reveals Differences Between Structural and Sequence-based Approaches
Source: Genomics Proteomics Bioinformatics. 2026 Feb 15;24(2):qzag014. doi: 10.1093/gpbjnl/qzag014 (PMC13472679; doi:10.1093/gpbjnl/qzag014)
Supplement: Supplementary file 1 [file qzag014_supplementary_data.zip › Figure S5.pdf]

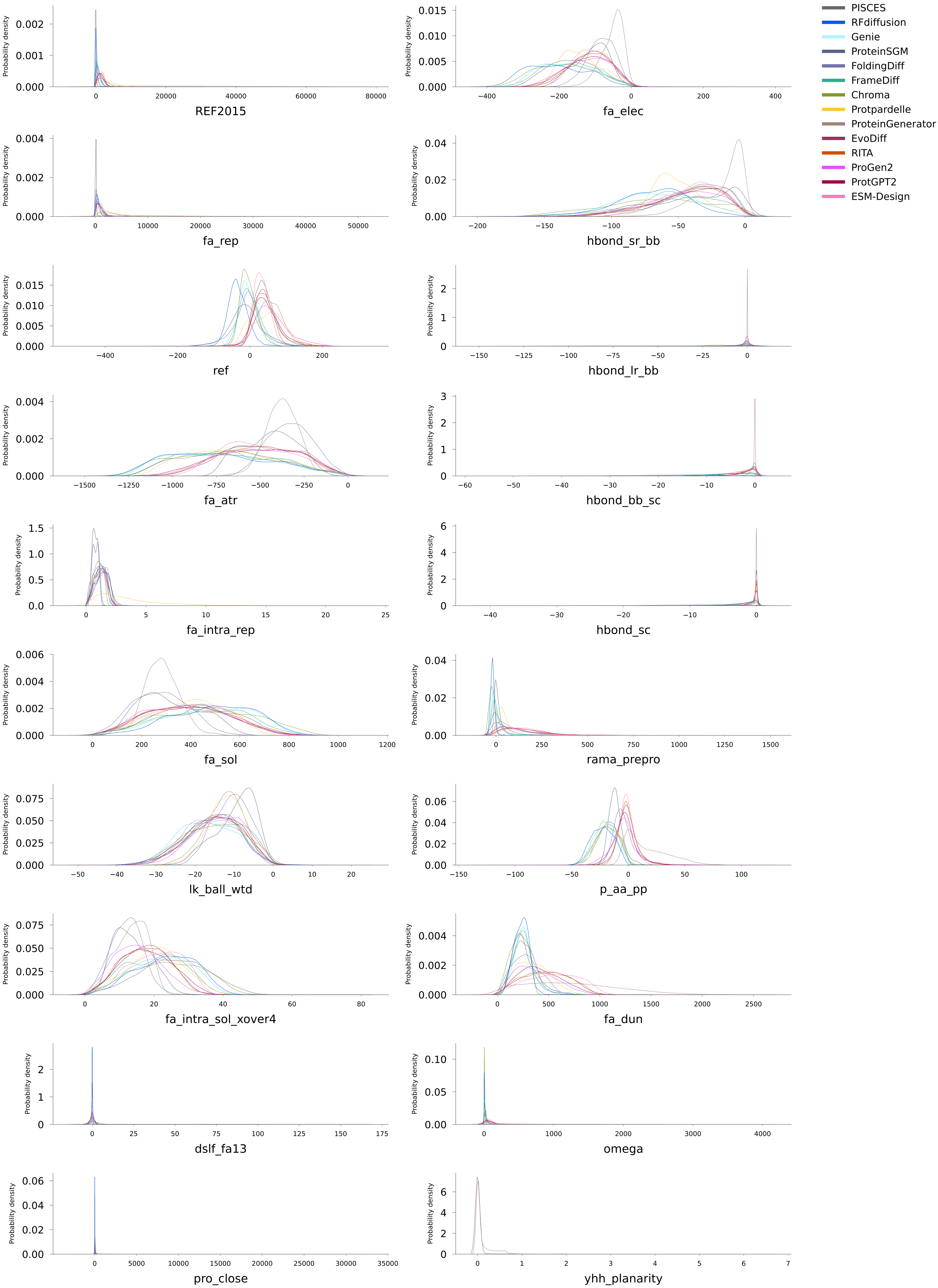

PISCES
RFdiffusion
Genie
ProteinSGM
FoldingDiff
FrameDiff
Chroma
Protpardelle
ProteinGenerator
EvoDiff
RITA
ProGen2
ProtGPT2
ESM-Design
Probability density
REF2015
fa_elec
fa_rep
hbond_sr_bb
ref
hbond_lr_bb
fa_atr
hbond_bb_sc
fa_intra_rep
hbond_sc
fa_sol
rama_prepro
lk_ball_wtd
p_aa_pp
fa_intra_sol_xover4
fa_dun
dslf_fa13
omega
pro_close
yhh_planarity
