# Supplementary material for: Benchmarking Generative AI Protein Models Reveals Differences Between Structural and Sequence-based Approaches
Source: Genomics Proteomics Bioinformatics. 2026 Feb 15;24(2):qzag014. doi: 10.1093/gpbjnl/qzag014 (PMC13472679; doi:10.1093/gpbjnl/qzag014)
Supplement: Supplementary file 1 [file qzag014_supplementary_data.zip › Figure S6.pdf]

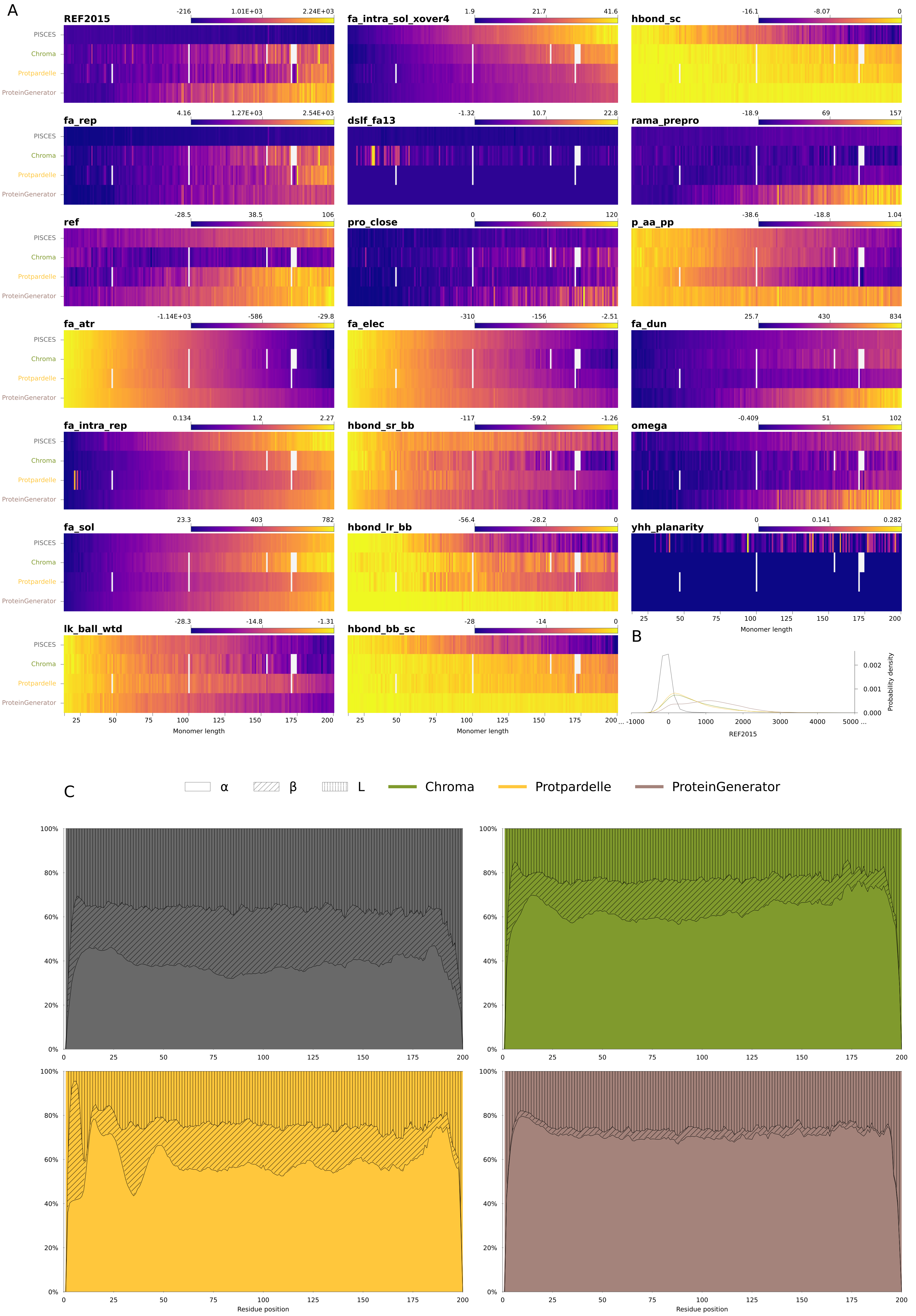
A
REF2015
fa_intra_sol_xover4
hbond_sc
fa_rep
dslf_fa13
rama_prepro
ref
pro_close
p_aa_pp
fa_atr
fa_elec
fa_dun
fa_intra_rep
hbond_sr_bb
omega
fa_sol
hbond_lr_bb
yhh_planarity
lk_ball_wtd
hbond_bb_sc
PISCES
Chroma
Protpardelle
ProteinGenerator
Monomer length
B
REF2015
Probability density
C
α
β
L
Chroma
Protpardelle
ProteinGenerator
Residue position
