# Supplementary material for: Benchmarking Generative AI Protein Models Reveals Differences Between Structural and Sequence-based Approaches
Source: Genomics Proteomics Bioinformatics. 2026 Feb 15;24(2):qzag014. doi: 10.1093/gpbjnl/qzag014 (PMC13472679; doi:10.1093/gpbjnl/qzag014)
Supplement: Supplementary file 1 [file qzag014_supplementary_data.zip › Figure S7.pdf]

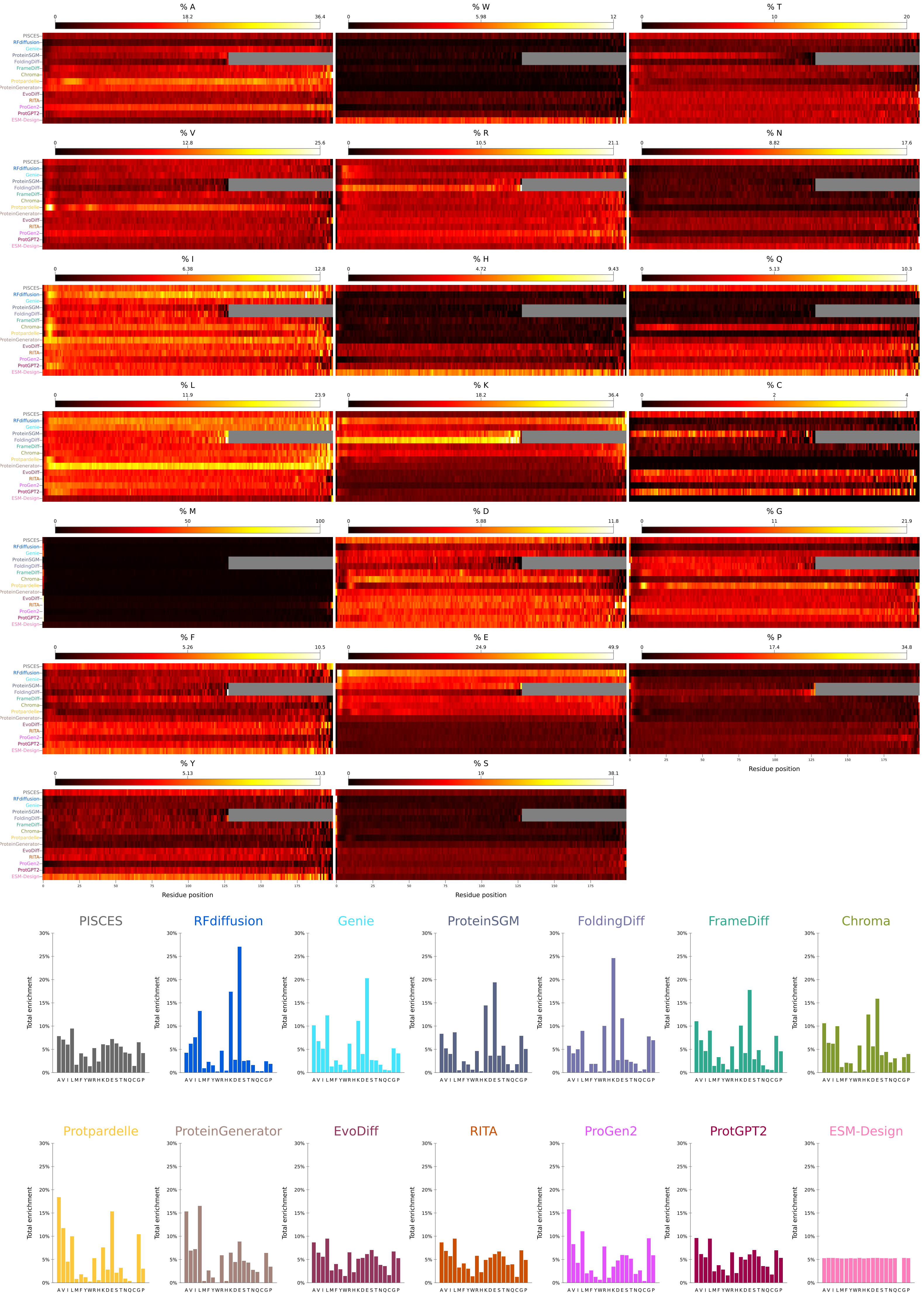

% A
0
18.2
36.4
% W
0
5.98
12
% T
0
10
20
% V
0
12.8
25.6
% R
0
10.5
21.1
% N
0
8.82
17.6
% I
0
6.38
12.8
% H
0
4.72
9.43
% Q
0
5.13
10.3
% L
0
11.9
23.9
% K
0
18.2
36.4
% C
0
2
4
% M
0
50
100
% D
0
5.88
11.8
% G
0
11
21.9
% F
0
5.26
10.5
% E
0
24.9
49.9
% P
0
17.4
34.8
% Y
0
5.13
10.3
% S
0
19
38.1
PISCES
RFdiffusion
Genie
ProteinSGM
FoldingDiff
FrameDiff
Chroma
Protpardelle
ProteinGenerator
EvoDiff
RITA
ProGen2
ProtGPT2
ESM-Design
0
25
50
75
100
125
150
175
Residue position
PISCES
RFdiffusion
Genie
ProteinSGM
FoldingDiff
FrameDiff
Chroma
Protpardelle
ProteinGenerator
EvoDiff
RITA
ProGen2
ProtGPT2
ESM-Design
Total enrichment
0%
5%
10%
15%
20%
25%
30%
A V I L M F Y W R H K D E S T N Q C G P
