# Supplementary material for: Benchmarking Generative AI Protein Models Reveals Differences Between Structural and Sequence-based Approaches
Source: Genomics Proteomics Bioinformatics. 2026 Feb 15;24(2):qzag014. doi: 10.1093/gpbjnl/qzag014 (PMC13472679; doi:10.1093/gpbjnl/qzag014)
Supplement: Supplementary file 1 [file qzag014_supplementary_data.zip › Figure S9.pdf]

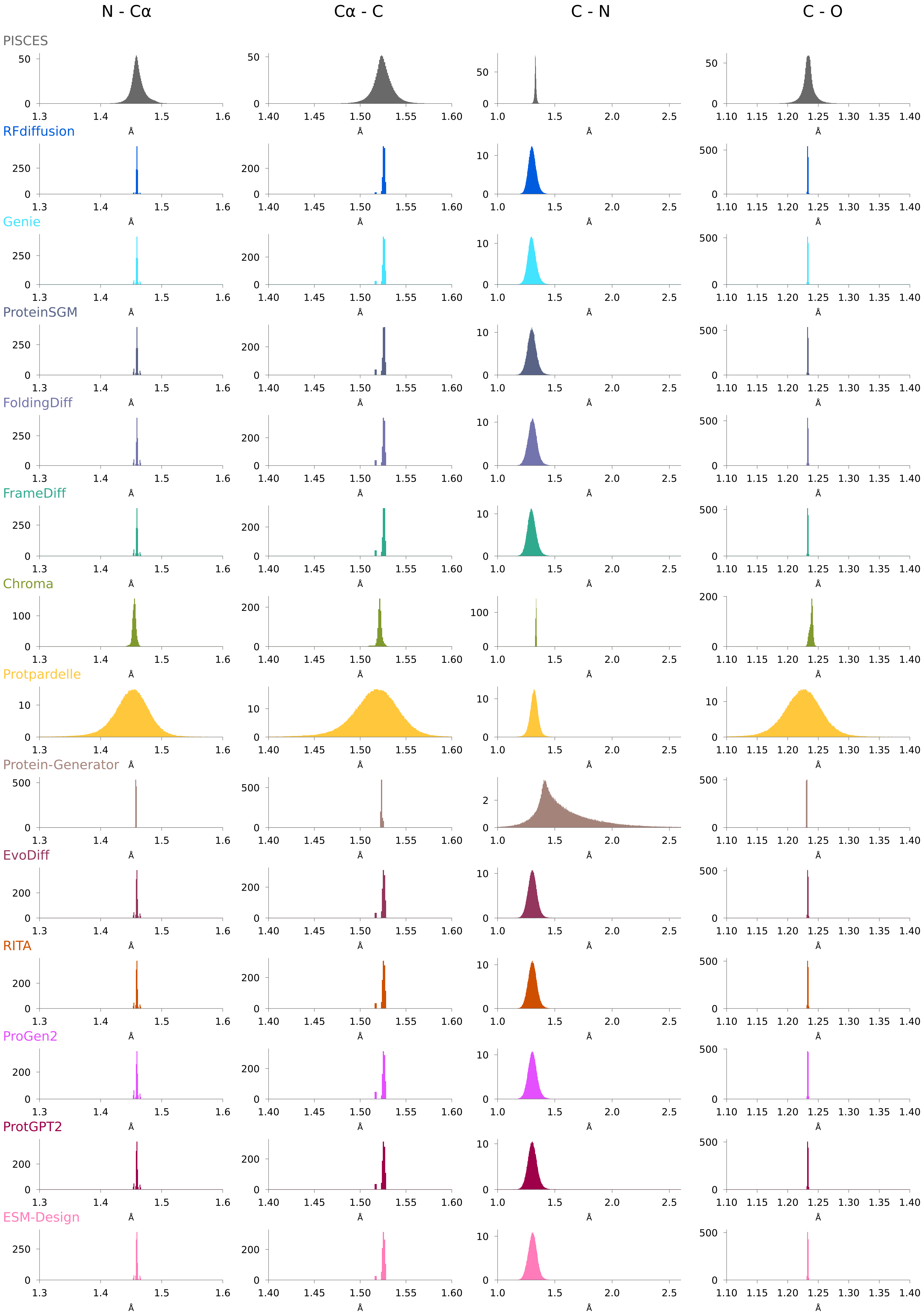
N - Cα
Cα - C
C - N
C - O
PISCES
RFdiffusion
Genie
ProteinSGM
FoldingDiff
FrameDiff
Chroma
Protpardelle
Protein-Generator
EvoDiff
RITA
ProGen2
ProtGPT2
ESM-Design
Å
1.3 1.4 1.5 1.6
1.40 1.45 1.50 1.55 1.60
1.0 1.5 2.0 2.5
1.10 1.15 1.20 1.25 1.30 1.35 1.40
