# Supplementary material for: Benchmarking Generative AI Protein Models Reveals Differences Between Structural and Sequence-based Approaches
Source: Genomics Proteomics Bioinformatics. 2026 Feb 15;24(2):qzag014. doi: 10.1093/gpbjnl/qzag014 (PMC13472679; doi:10.1093/gpbjnl/qzag014)
Supplement: Supplementary file 1 [file qzag014_supplementary_data.zip › Figure S10.pdf]

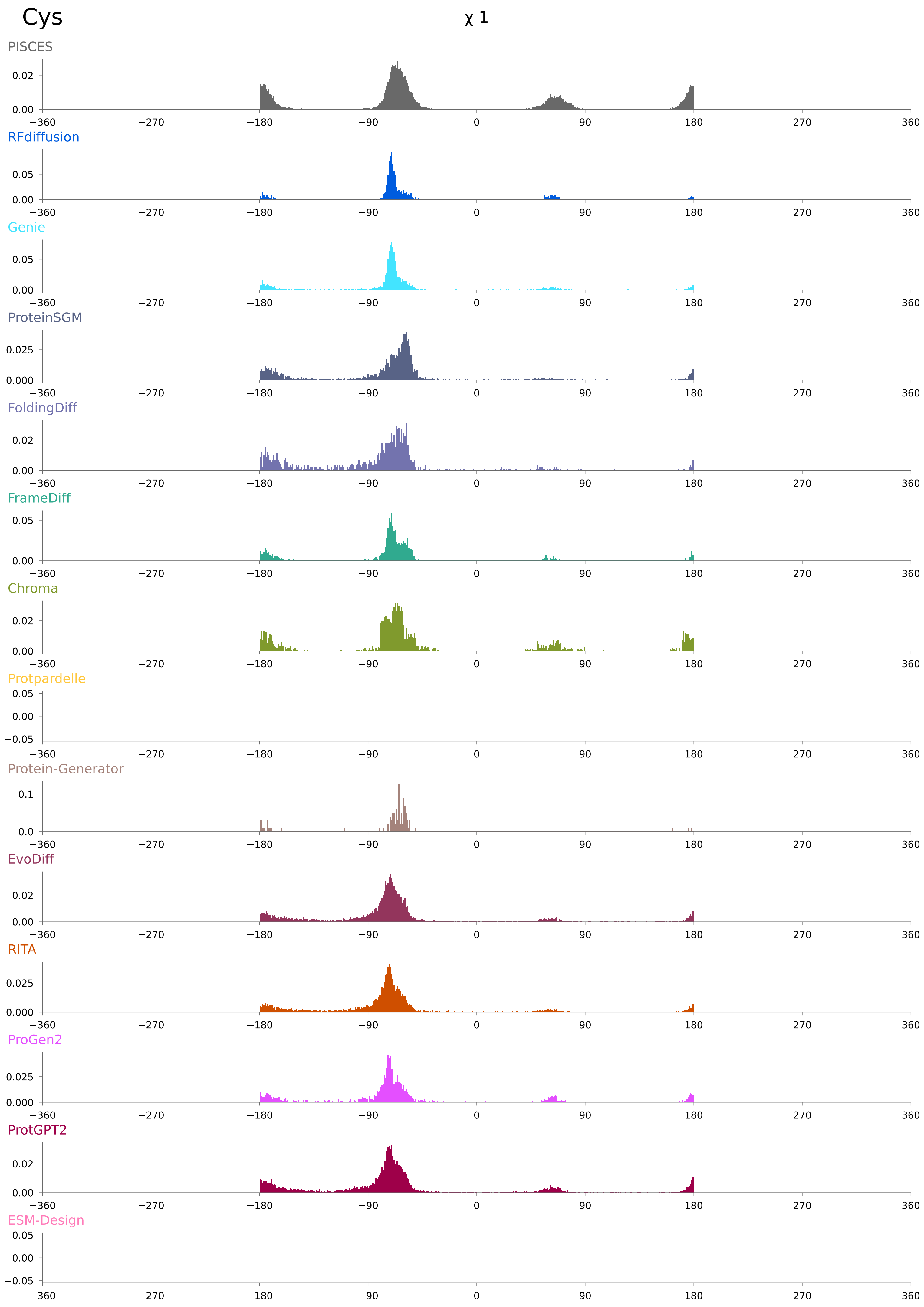
Cys
χ 1
PISCES
RFdiffusion
Genie
ProteinSGM
FoldingDiff
FrameDiff
Chroma
Protpardelle
Protein-Generator
EvoDiff
RITA
ProGen2
ProtGPT2
ESM-Design
−360
−270
−180
−90
0
90
180
270
360

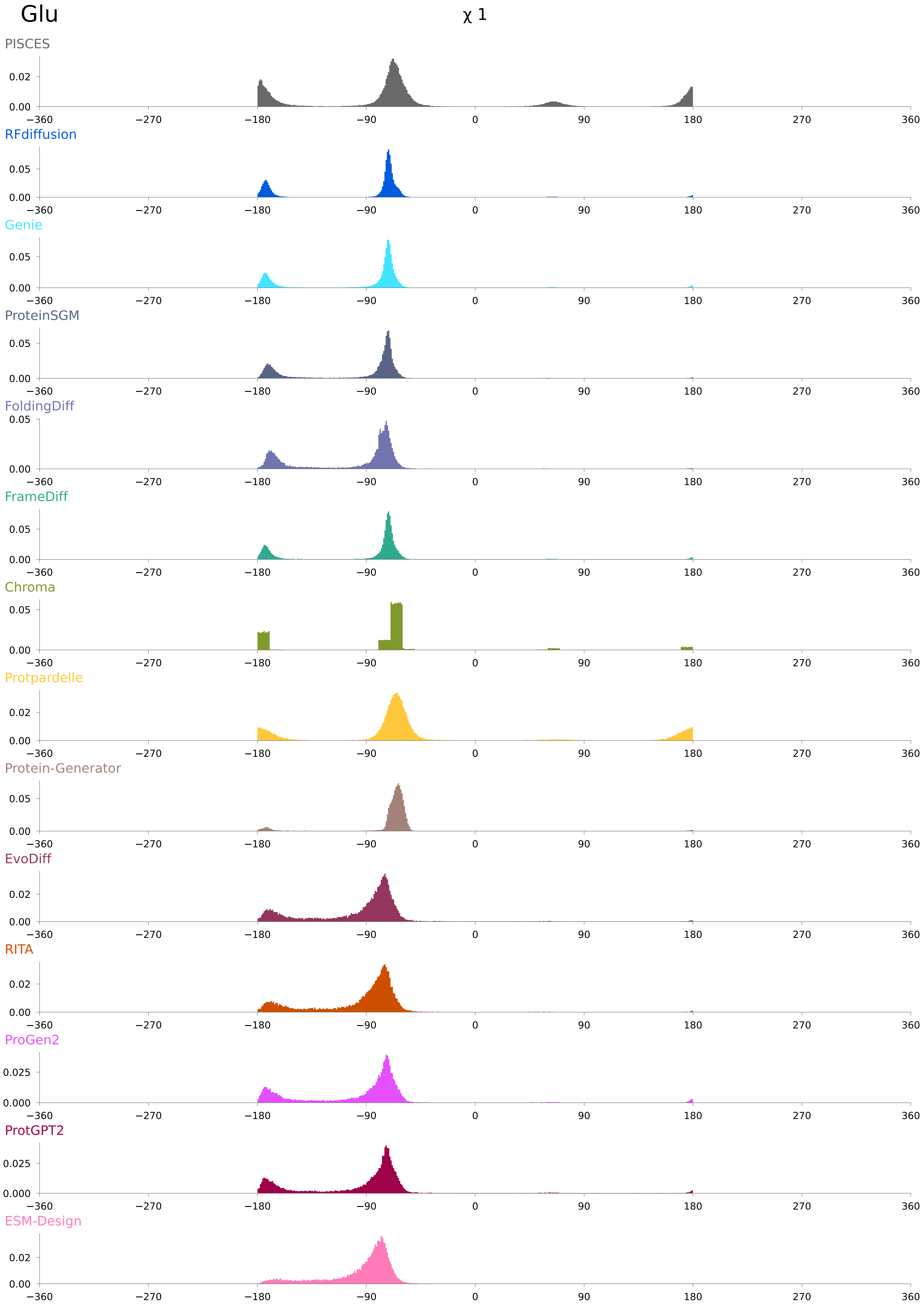
Glu
χ 1
PISCES
RFdiffusion
Genie
ProteinSGM
FoldingDiff
FrameDiff
Chroma
Protpardelle
Protein-Generator
EvoDiff
RITA
ProGen2
ProtGPT2
ESM-Design
−360
−270
−180
−90
0
90
180
270
360

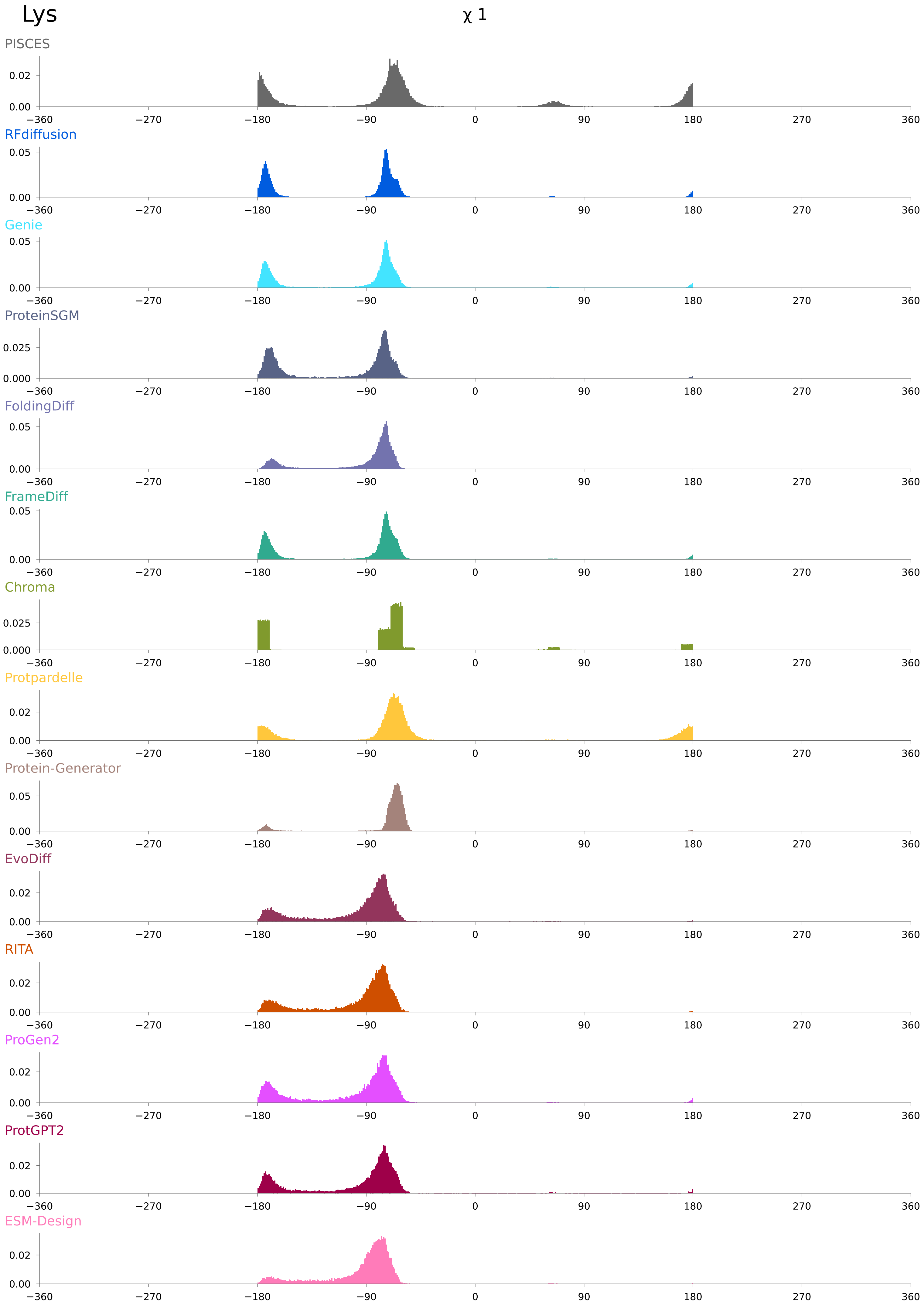
Lys
χ 1
PISCES
0.02
0.00
RFdiffusion
0.05
0.00
Genie
0.05
0.00
ProteinSGM
0.025
0.000
FoldingDiff
0.05
0.00
FrameDiff
0.05
0.00
Chroma
0.025
0.000
Protpardelle
0.02
0.00
Protein-Generator
0.05
0.00
EvoDiff
0.02
0.00
RITA
0.02
0.00
ProGen2
0.02
0.00
ProtGPT2
0.02
0.00
ESM-Design
0.02
0.00
−360 −270 −180 −90 0 90 180 270 360

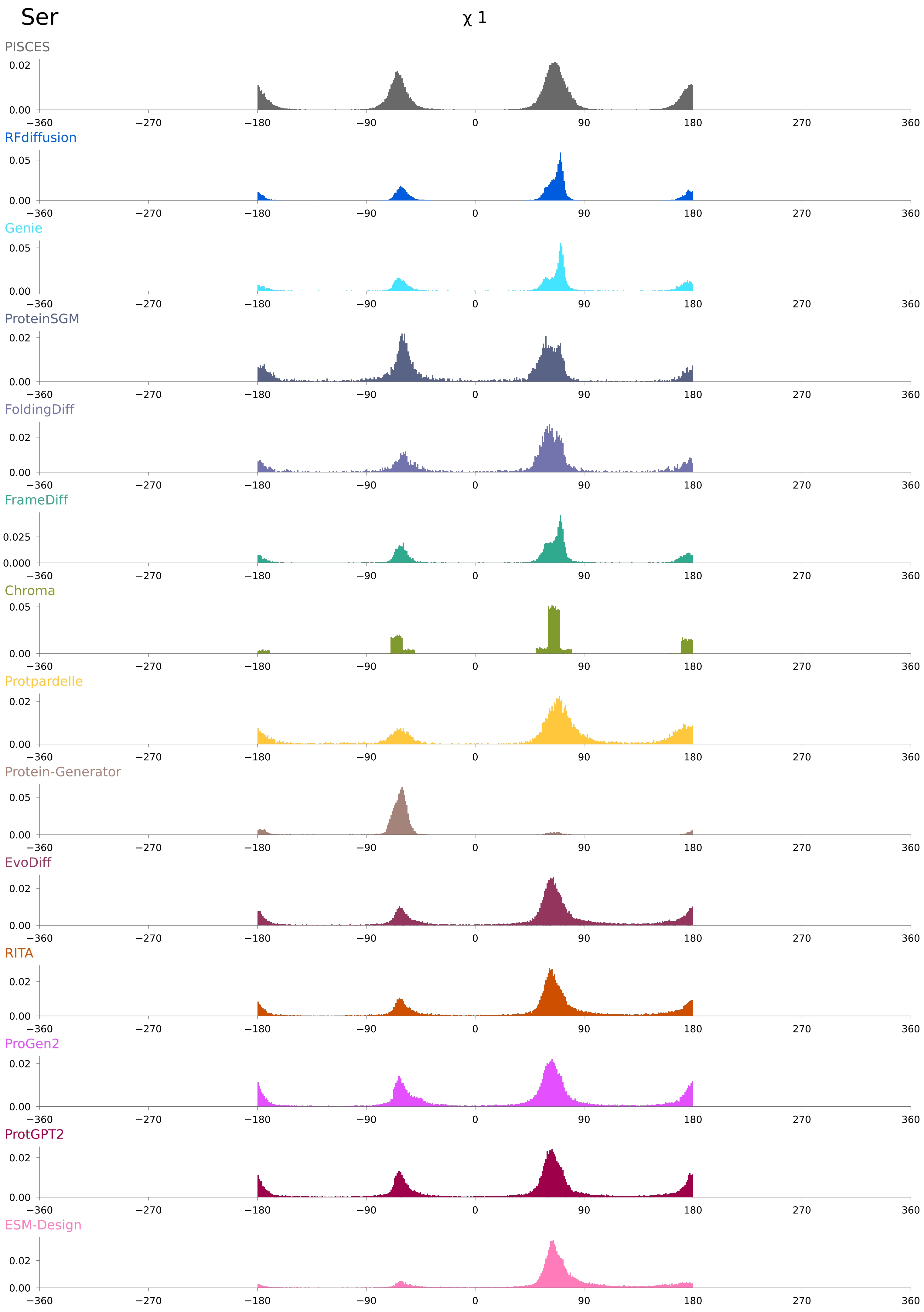

Ser
χ 1
PISCES
0.02
0.00
−360 −270 −180 −90 0 90 180 270 360
RFdiffusion
0.05
0.00
−360 −270 −180 −90 0 90 180 270 360
Genie
0.05
0.00
−360 −270 −180 −90 0 90 180 270 360
ProteinSGM
0.02
0.00
−360 −270 −180 −90 0 90 180 270 360
FoldingDiff
0.02
0.00
−360 −270 −180 −90 0 90 180 270 360
FrameDiff
0.025
0.000
−360 −270 −180 −90 0 90 180 270 360
Chroma
0.05
0.00
−360 −270 −180 −90 0 90 180 270 360
Protpardelle
0.02
0.00
−360 −270 −180 −90 0 90 180 270 360
Protein-Generator
0.05
0.00
−360 −270 −180 −90 0 90 180 270 360
EvoDiff
0.02
0.00
−360 −270 −180 −90 0 90 180 270 360
RITA
0.02
0.00
−360 −270 −180 −90 0 90 180 270 360
ProGen2
0.02
0.00
−360 −270 −180 −90 0 90 180 270 360
ProtGPT2
0.02
0.00
−360 −270 −180 −90 0 90 180 270 360
ESM-Design
0.02
0.00
−360 −270 −180 −90 0 90 180 270 360

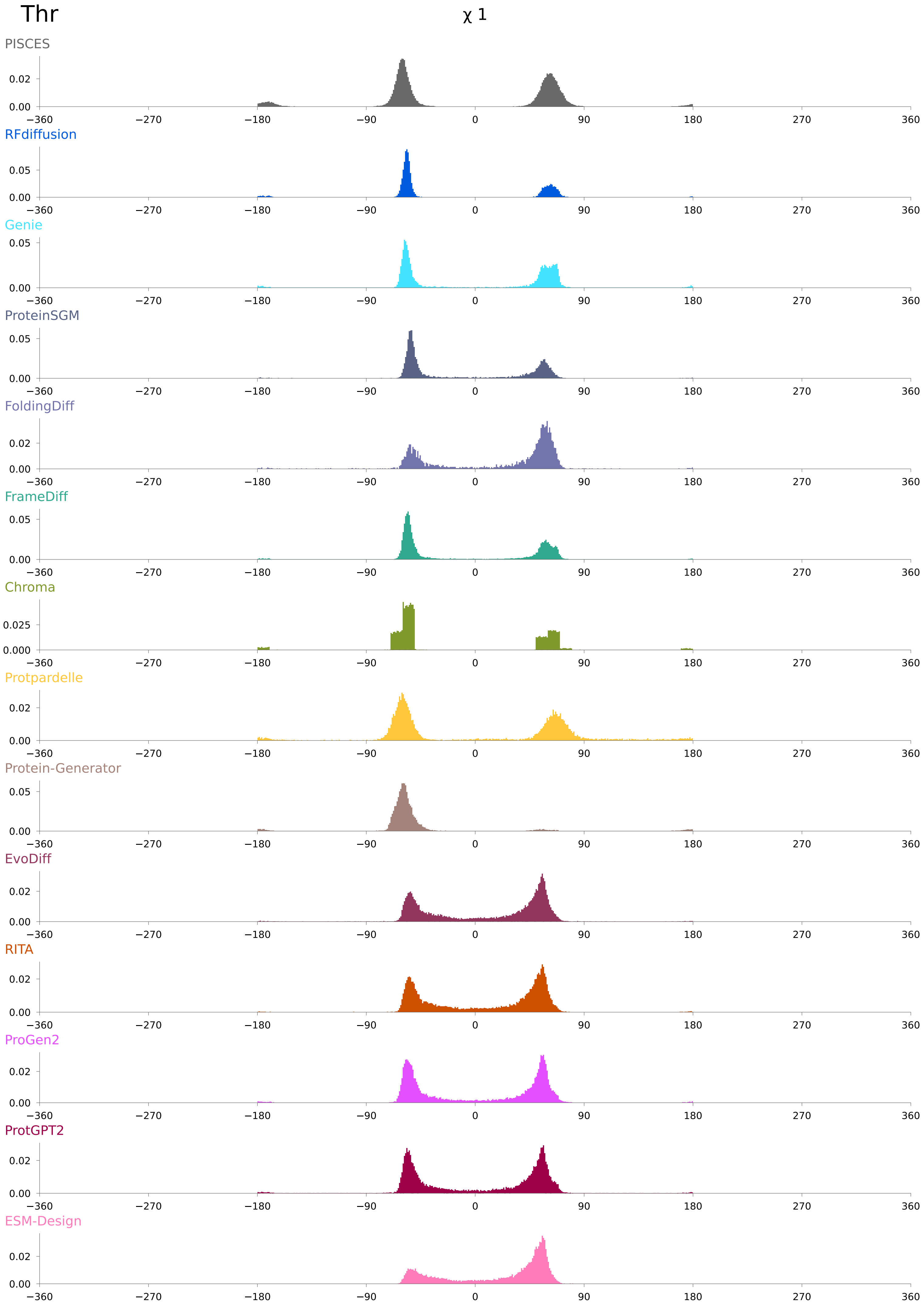

Thr
χ 1
PISCES
RFdiffusion
Genie
ProteinSGM
FoldingDiff
FrameDiff
Chroma
Protpardelle
Protein-Generator
EvoDiff
RITA
ProGen2
ProtGPT2
ESM-Design
−360
−270
−180
−90
0
90
180
270
360

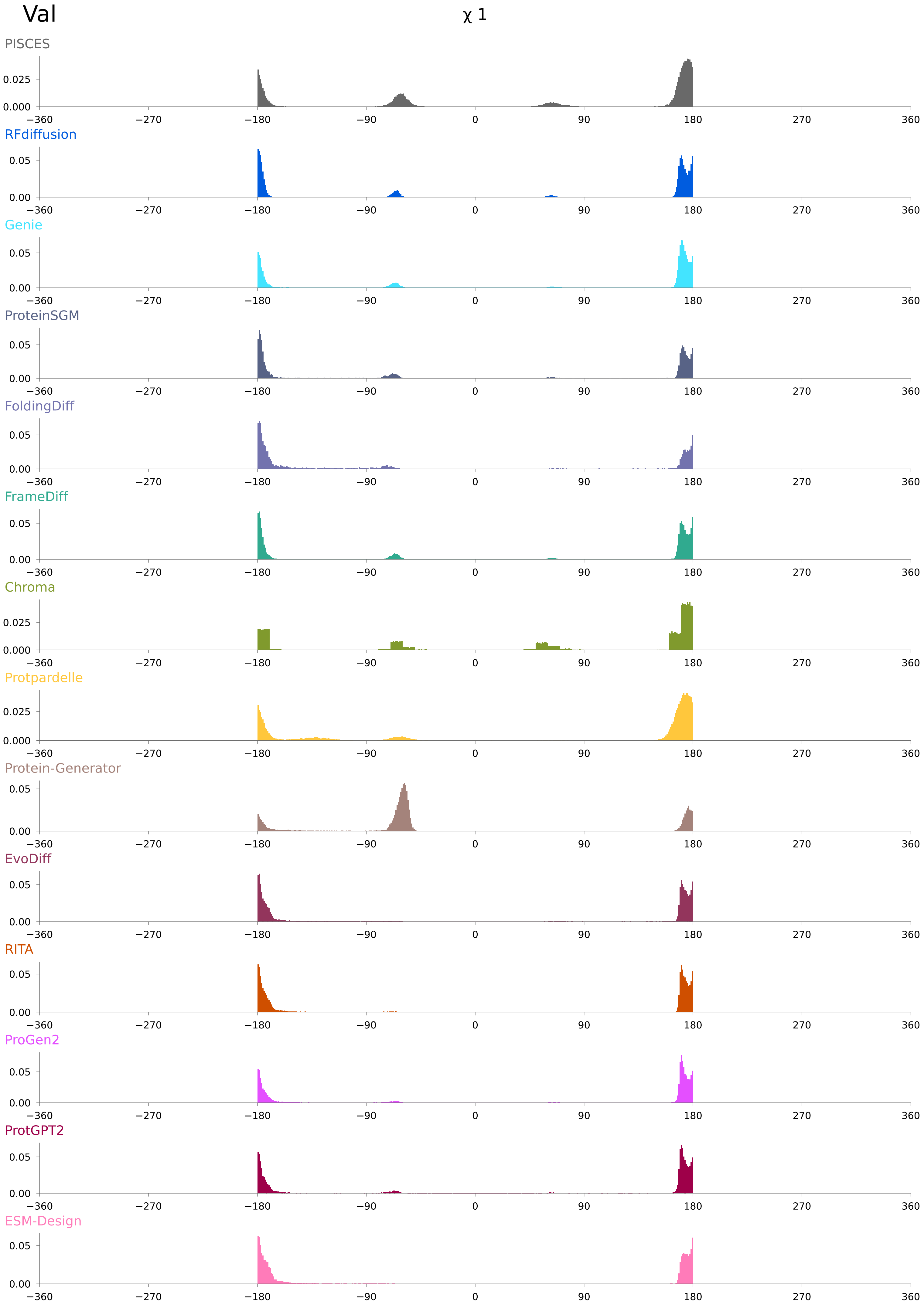
Val
χ 1
PISCES
RFdiffusion
Genie
ProteinSGM
FoldingDiff
FrameDiff
Chroma
Protpardelle
Protein-Generator
EvoDiff
RITA
ProGen2
ProtGPT2
ESM-Design
0.025
0.000
0.05
0.00
−360
−270
−180
−90
0
90
180
270
360

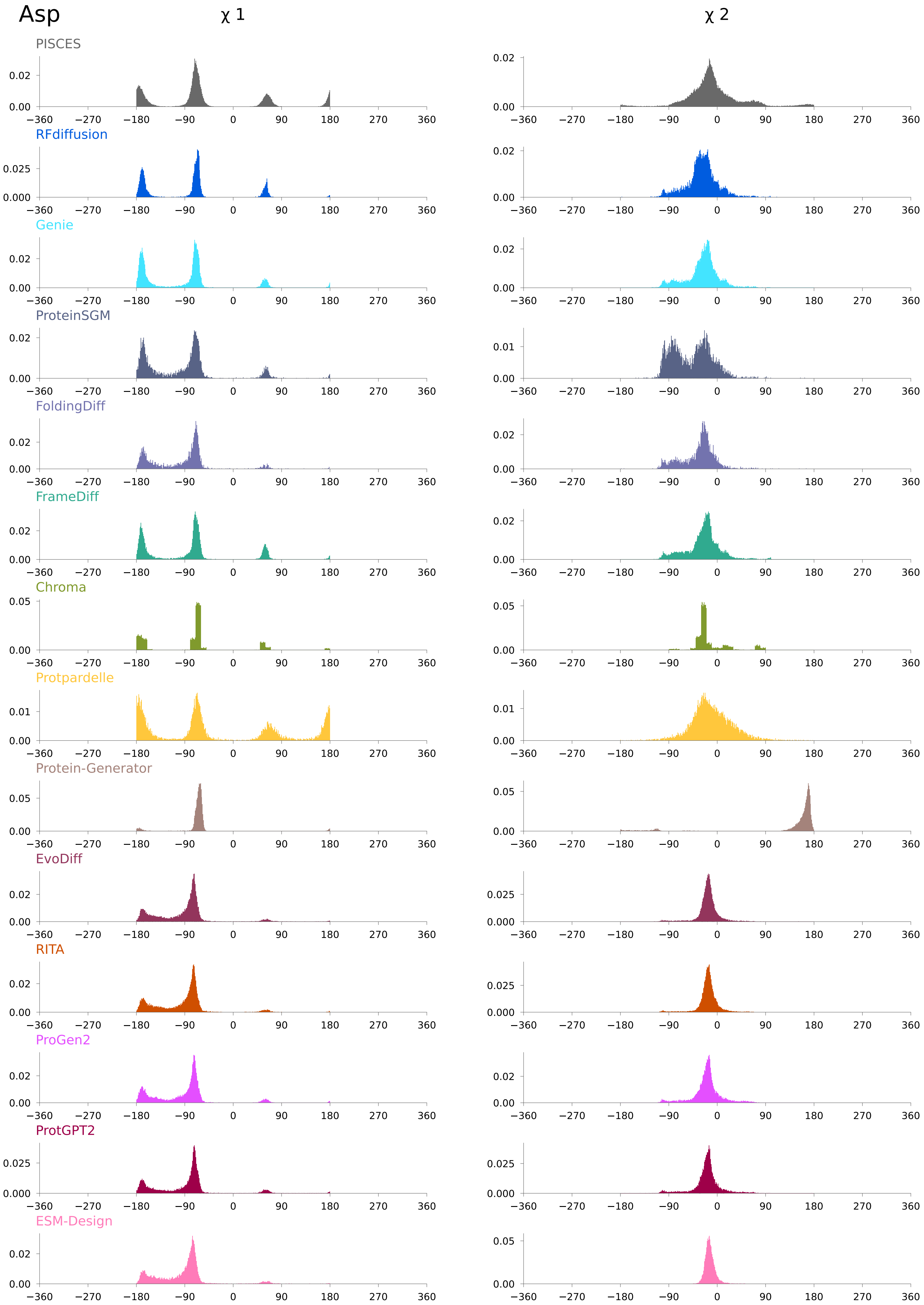
Asp
χ 1
χ 2
PISCES
RFdiffusion
Genie
ProteinSGM
FoldingDiff
FrameDiff
Chroma
Protpardelle
Protein-Generator
EvoDiff
RITA
ProGen2
ProtGPT2
ESM-Design

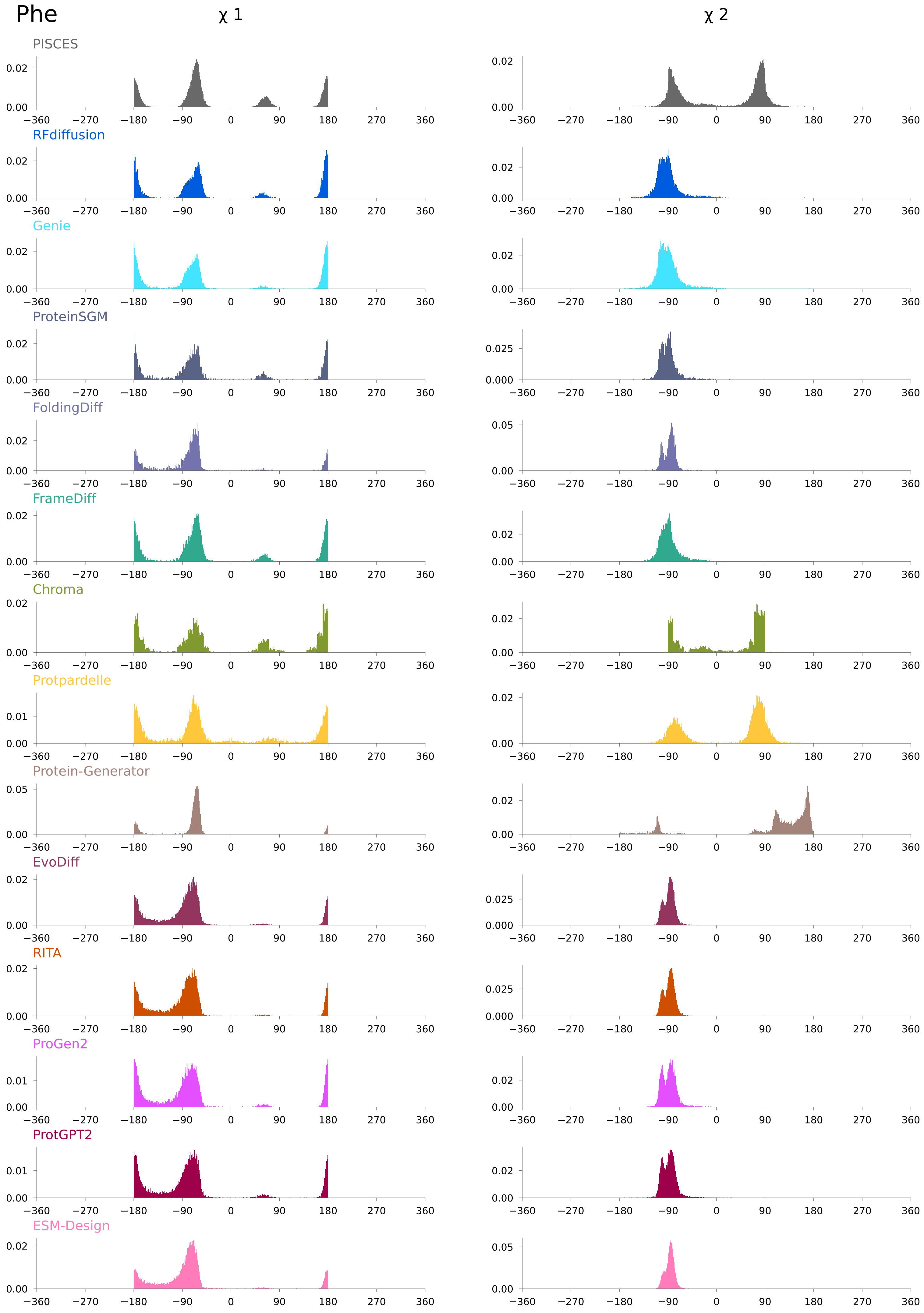
Phe
χ 1
χ 2
PISCES
RFdiffusion
Genie
ProteinSGM
FoldingDiff
FrameDiff
Chroma
Protpardelle
Protein-Generator
EvoDiff
RITA
ProGen2
ProtGPT2
ESM-Design
−360
−270
−180
−90
0
90
180
270
360
0.00
0.01
0.02
0.05
0.000
0.025

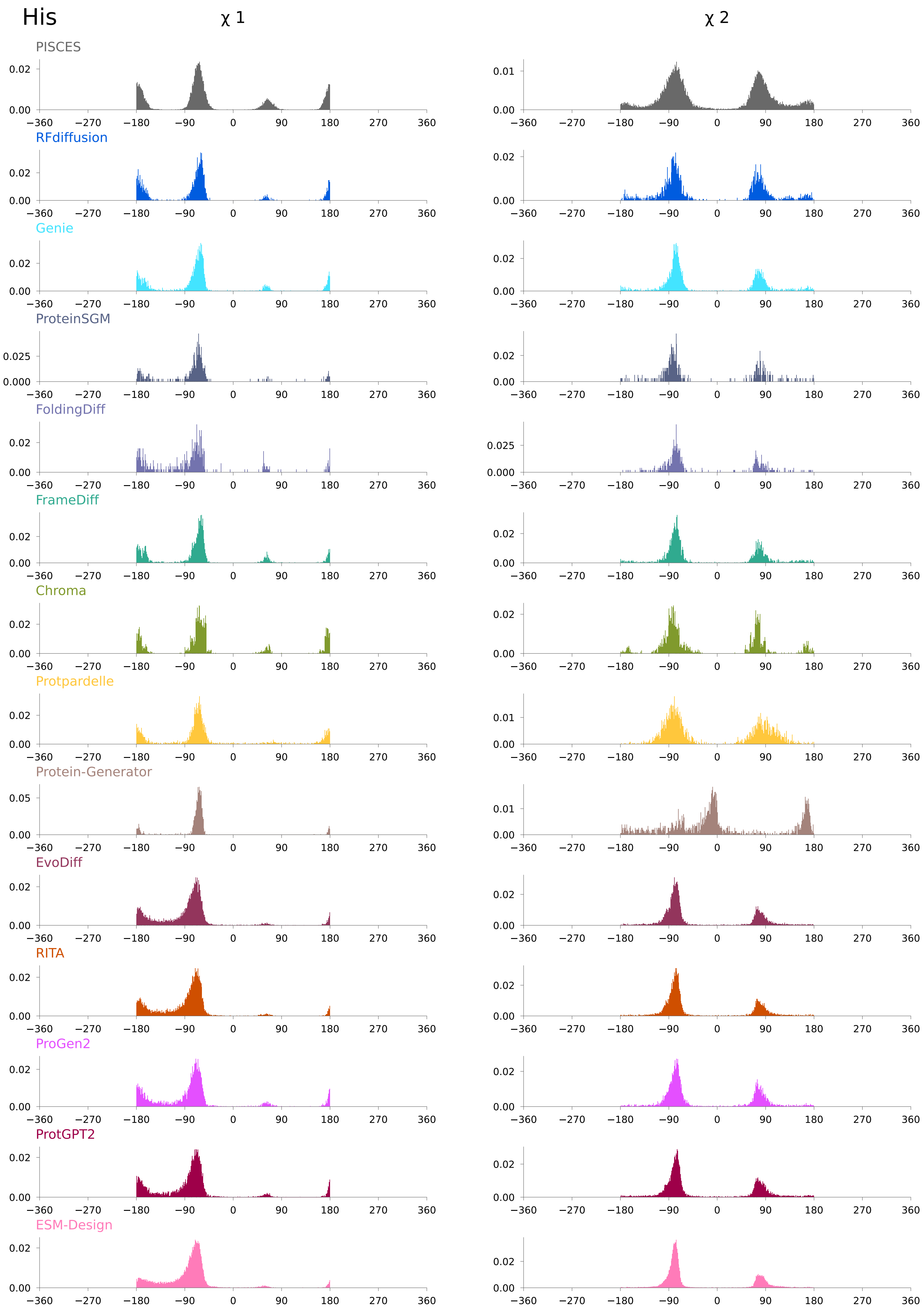
His
χ 1
χ 2
PISCES
RFdiffusion
Genie
ProteinSGM
FoldingDiff
FrameDiff
Chroma
Protpardelle
Protein-Generator
EvoDiff
RITA
ProGen2
ProtGPT2
ESM-Design
−360 −270 −180 −90 0 90 180 270 360

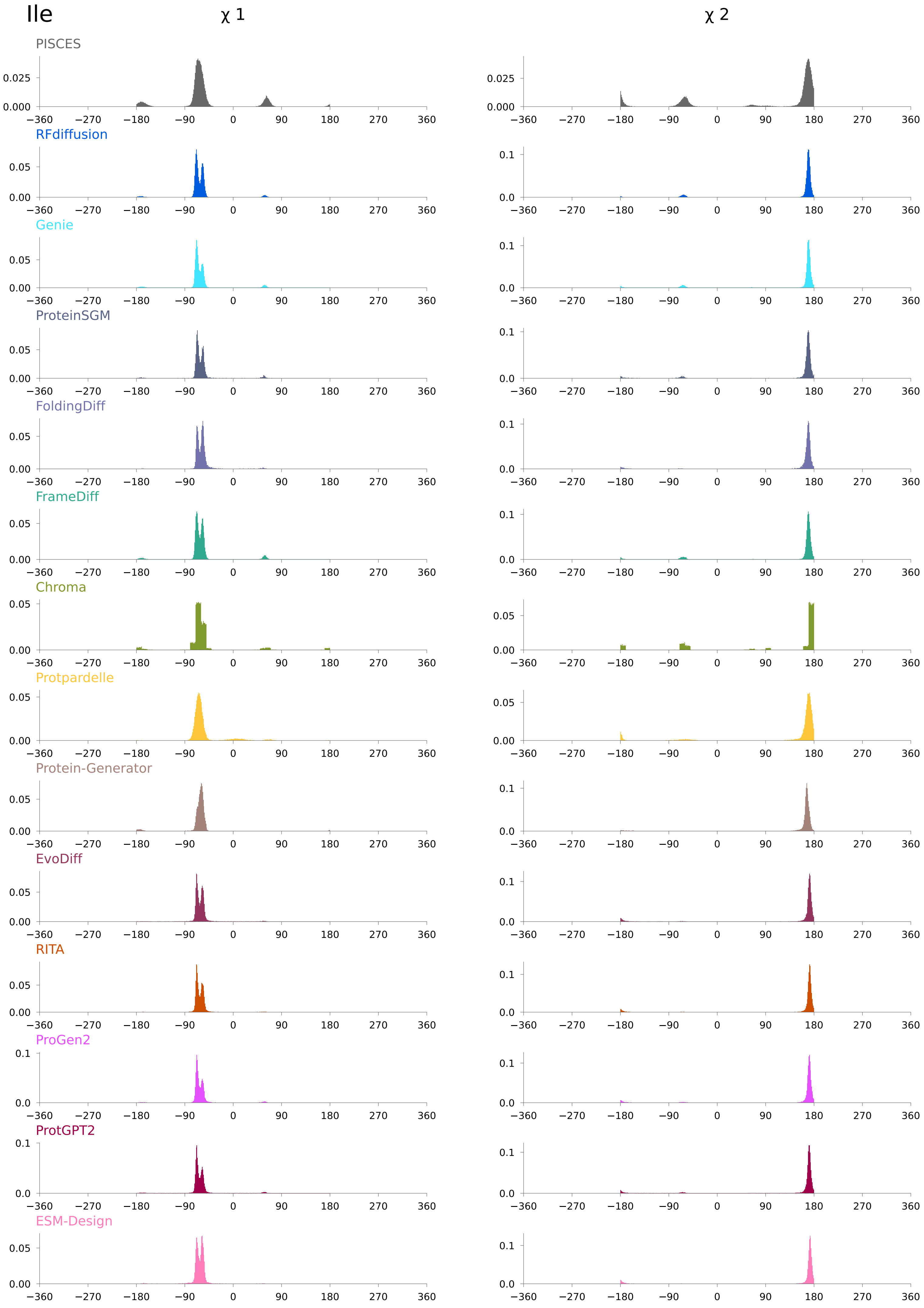
Ile
χ 1
χ 2
PISCES
RFdiffusion
Genie
ProteinSGM
FoldingDiff
FrameDiff
Chroma
Protpardelle
Protein-Generator
EvoDiff
RITA
ProGen2
ProtGPT2
ESM-Design
0.025
0.000
0.05
0.00
0.1
0.0
−360
−270
−180
−90
0
90
180
270
360

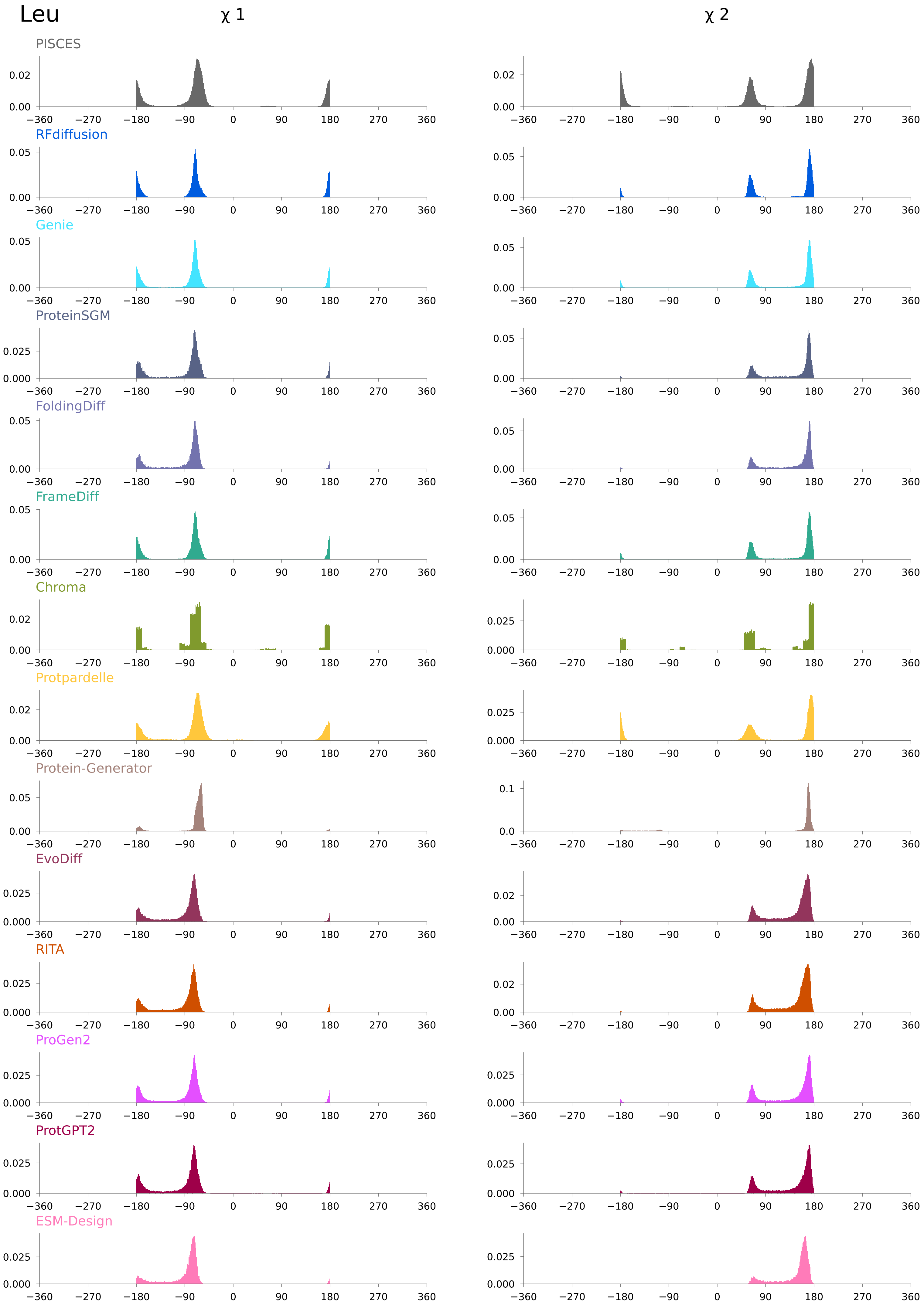

Leu
χ 1
χ 2
PISCES
RFdiffusion
Genie
ProteinSGM
FoldingDiff
FrameDiff
Chroma
Protpardelle
Protein-Generator
EvoDiff
RITA
ProGen2
ProtGPT2
ESM-Design
−360 −270 −180 −90 0 90 180 270 360

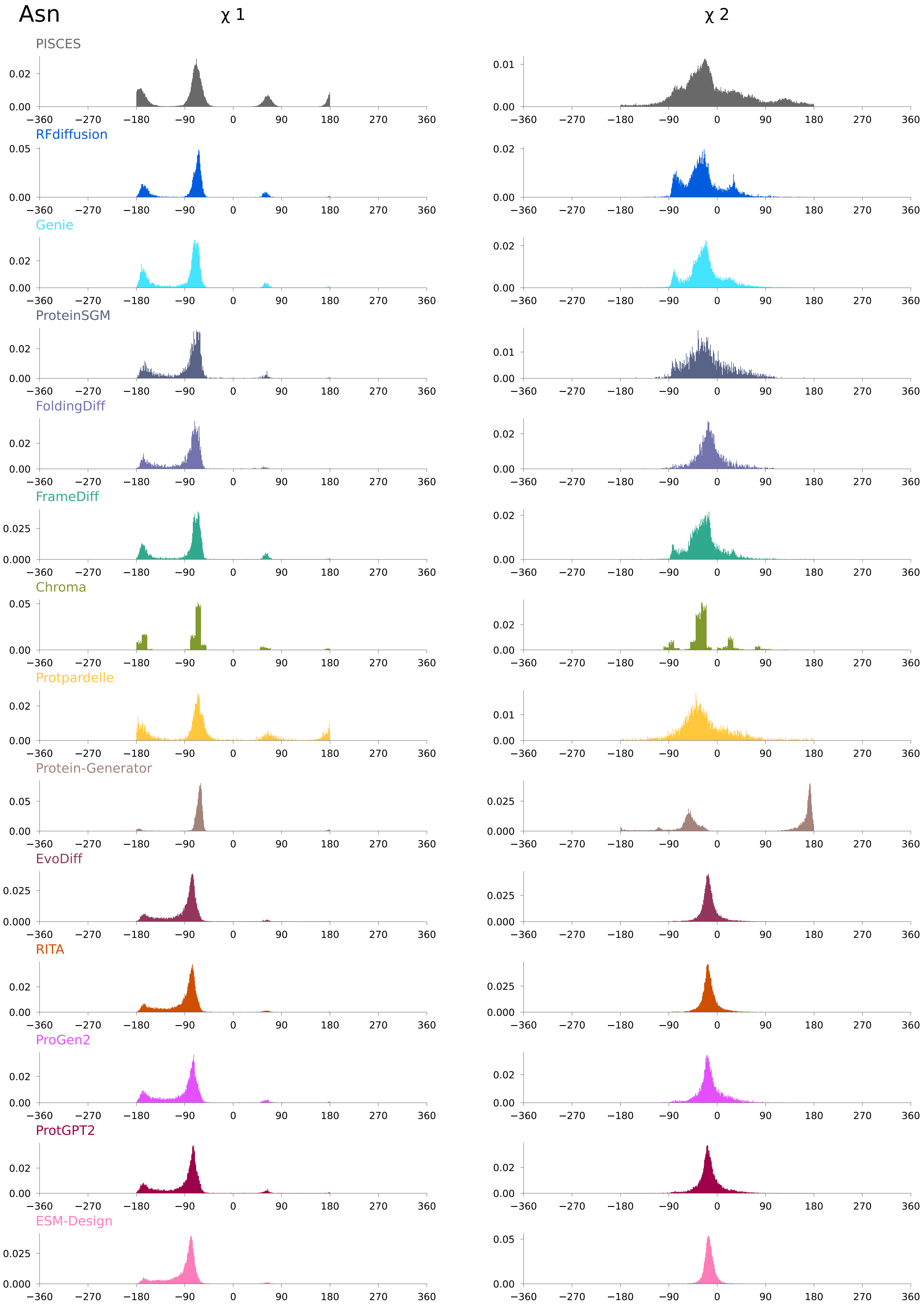
Asn
χ 1
χ 2
PISCES
RFdiffusion
Genie
ProteinSGM
FoldingDiff
FrameDiff
Chroma
Protpardelle
Protein-Generator
EvoDiff
RITA
ProGen2
ProtGPT2
ESM-Design
−360
−270
−180
−90
0
90
180
270
360

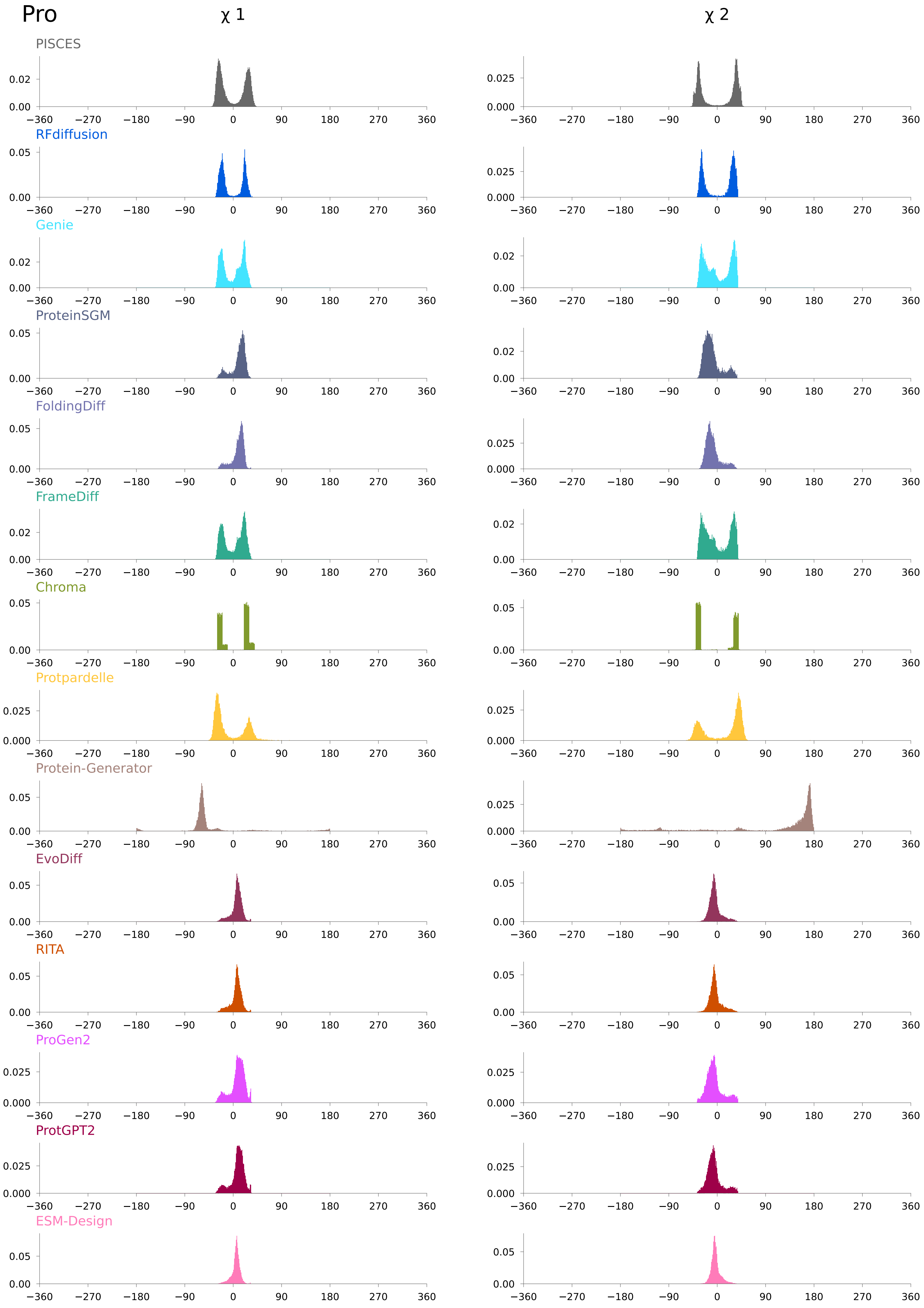

Pro
χ 1
χ 2
PISCES
RFdiffusion
Genie
ProteinSGM
FoldingDiff
FrameDiff
Chroma
Protpardelle
Protein-Generator
EvoDiff
RITA
ProGen2
ProtGPT2
ESM-Design

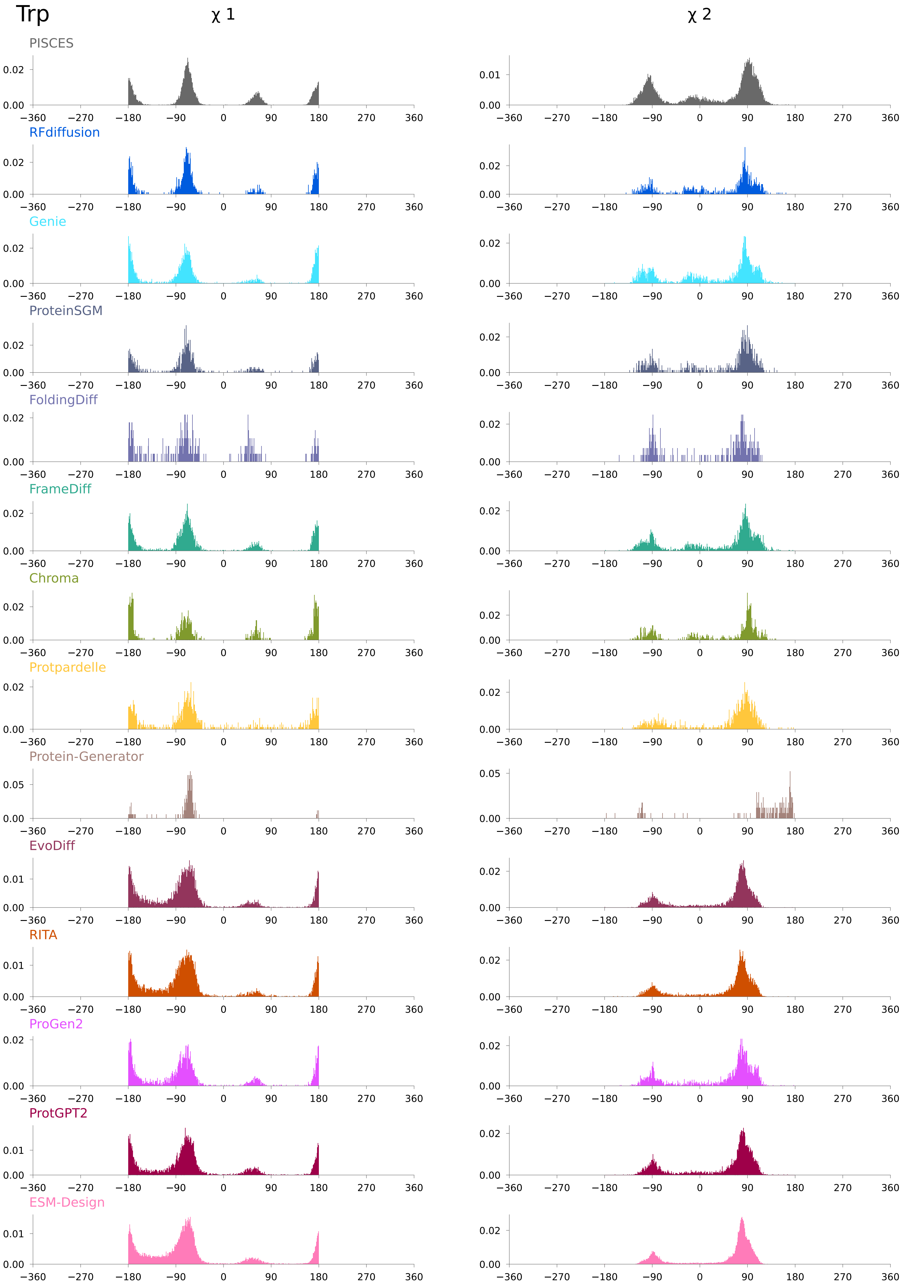
Trp
χ 1
χ 2
PISCES
RFdiffusion
Genie
ProteinSGM
FoldingDiff
FrameDiff
Chroma
Protpardelle
Protein-Generator
EvoDiff
RITA
ProGen2
ProtGPT2
ESM-Design
−360 −270 −180 −90 0 90 180 270 360
0.00
0.01
0.02
0.05

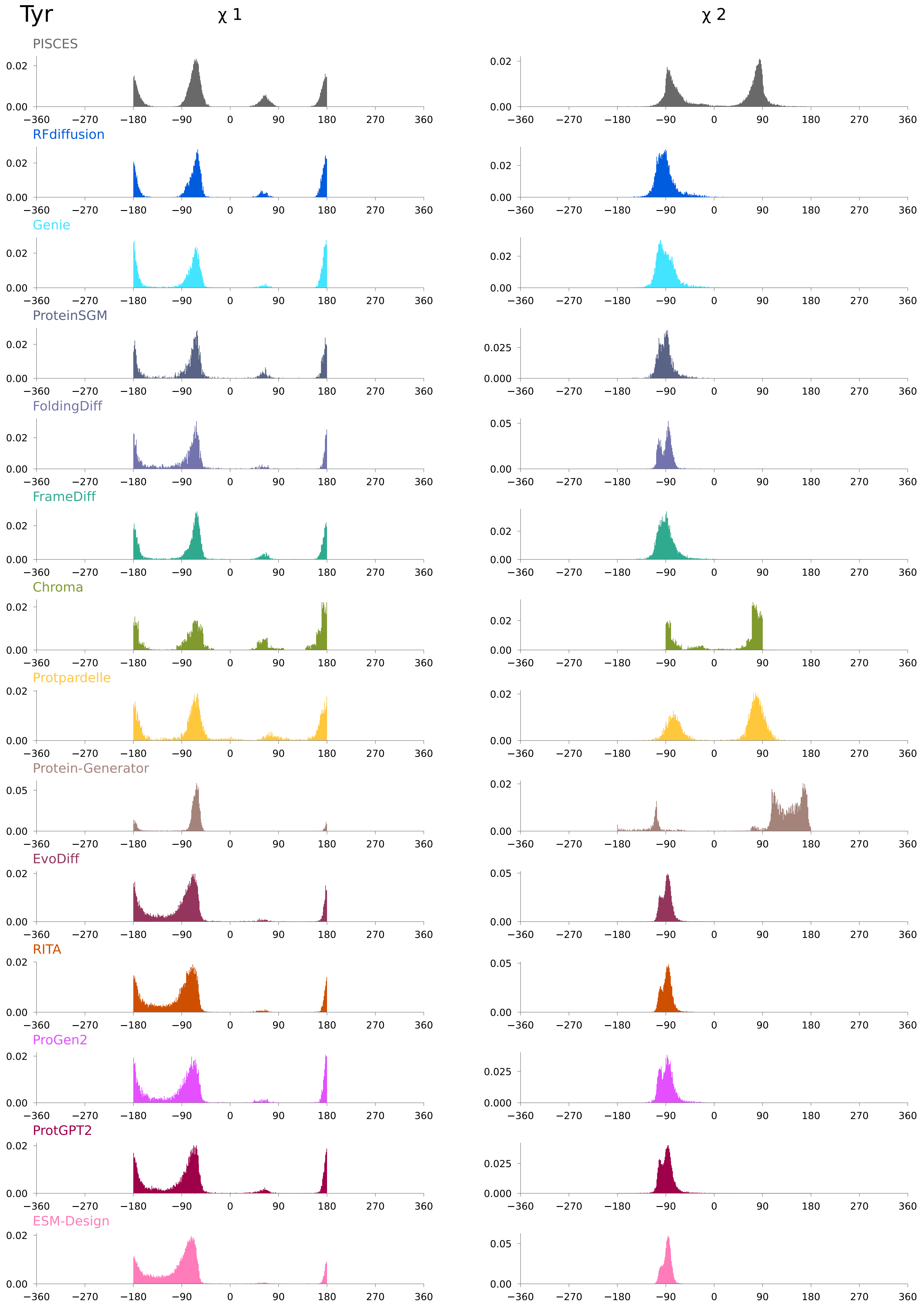
Tyr
χ 1
χ 2
PISCES
RFdiffusion
Genie
ProteinSGM
FoldingDiff
FrameDiff
Chroma
Protpardelle
Protein-Generator
EvoDiff
RITA
ProGen2
ProtGPT2
ESM-Design
0.00
0.02
0.025
0.05
−360
−270
−180
−90
0
90
180
270
360

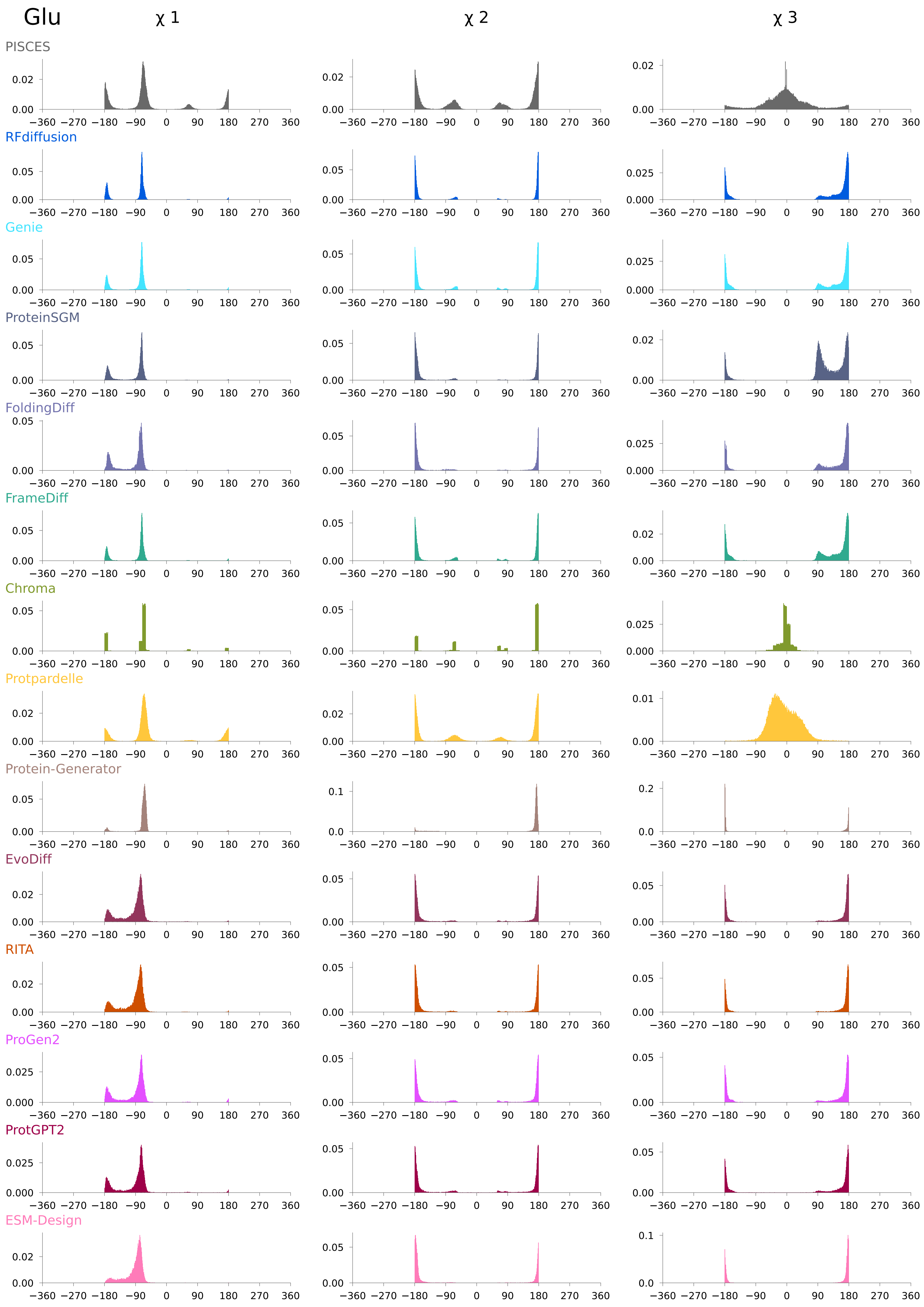
Glu
χ 1
χ 2
χ 3
PISCES
RFdiffusion
Genie
ProteinSGM
FoldingDiff
FrameDiff
Chroma
Protpardelle
Protein-Generator
EvoDiff
RITA
ProGen2
ProtGPT2
ESM-Design

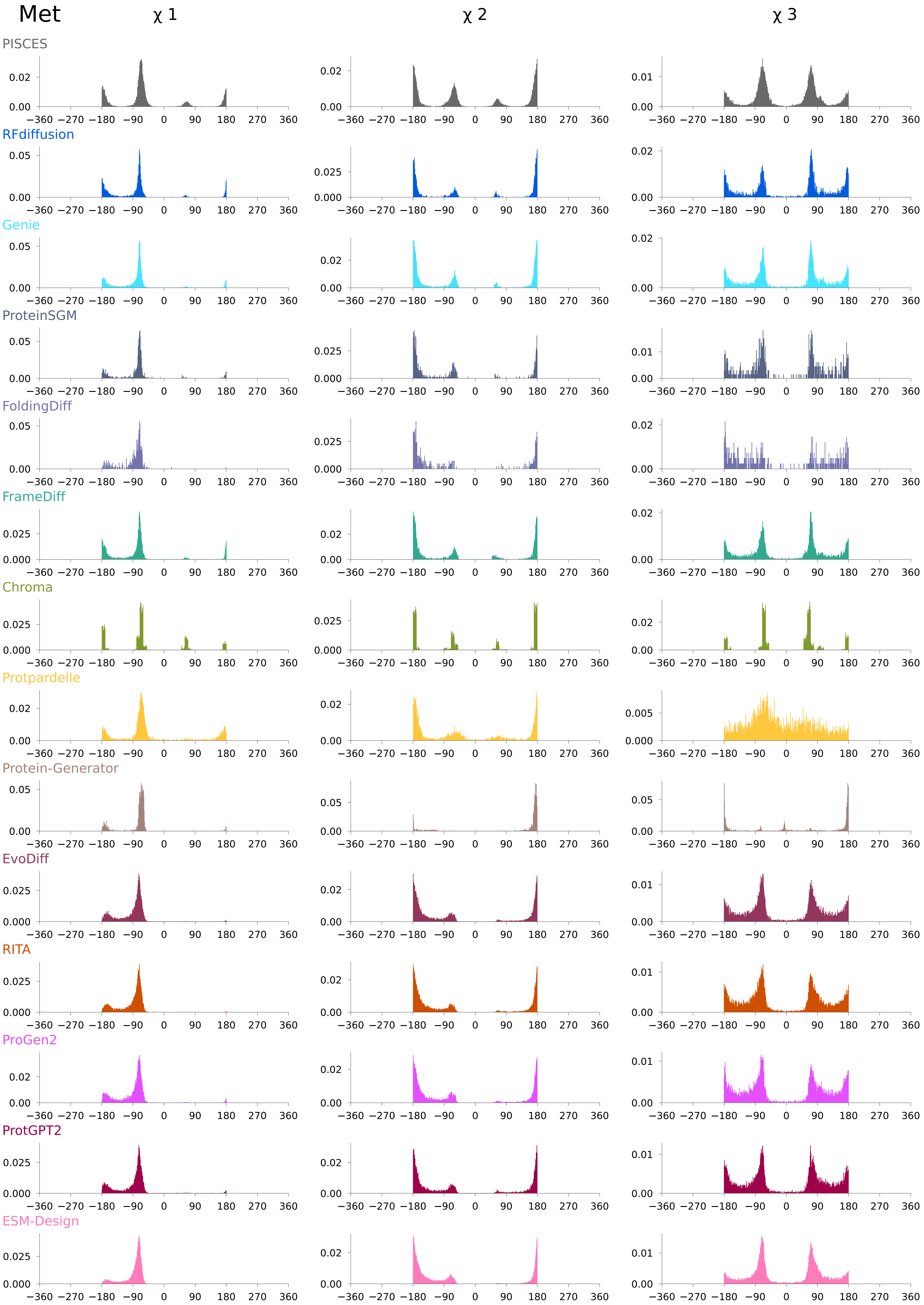
Met
χ 1
χ 2
χ 3
PISCES
RFdiffusion
Genie
ProteinSGM
FoldingDiff
FrameDiff
Chroma
Protpardelle
Protein-Generator
EvoDiff
RITA
ProGen2
ProtGPT2
ESM-Design
−360 −270 −180 −90 0 90 180 270 360

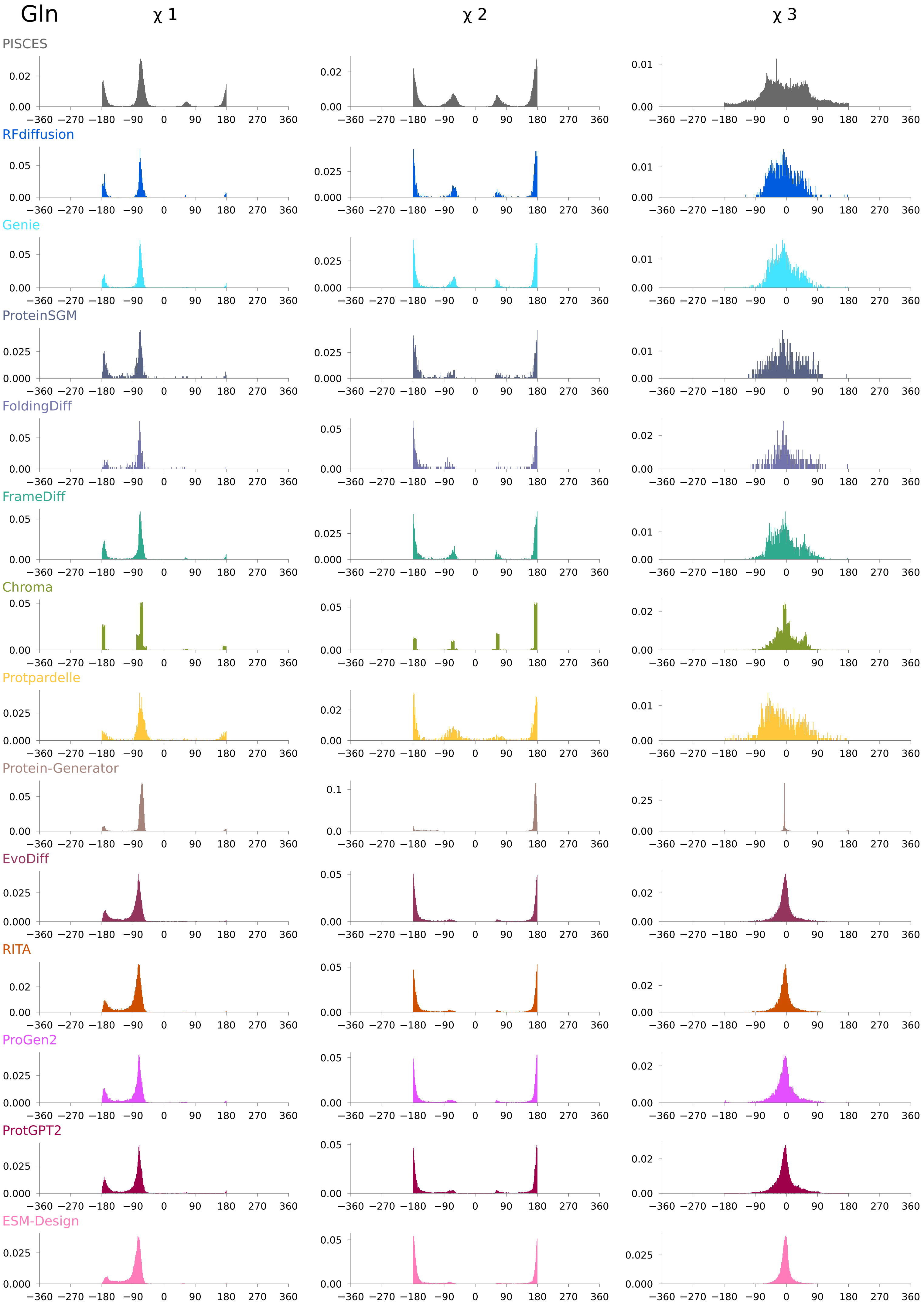
Gln
χ 1
χ 2
χ 3
PISCES
RFdiffusion
Genie
ProteinSGM
FoldingDiff
FrameDiff
Chroma
Protpardelle
Protein-Generator
EvoDiff
RITA
ProGen2
ProtGPT2
ESM-Design
−360 −270 −180 −90 0 90 180 270 360

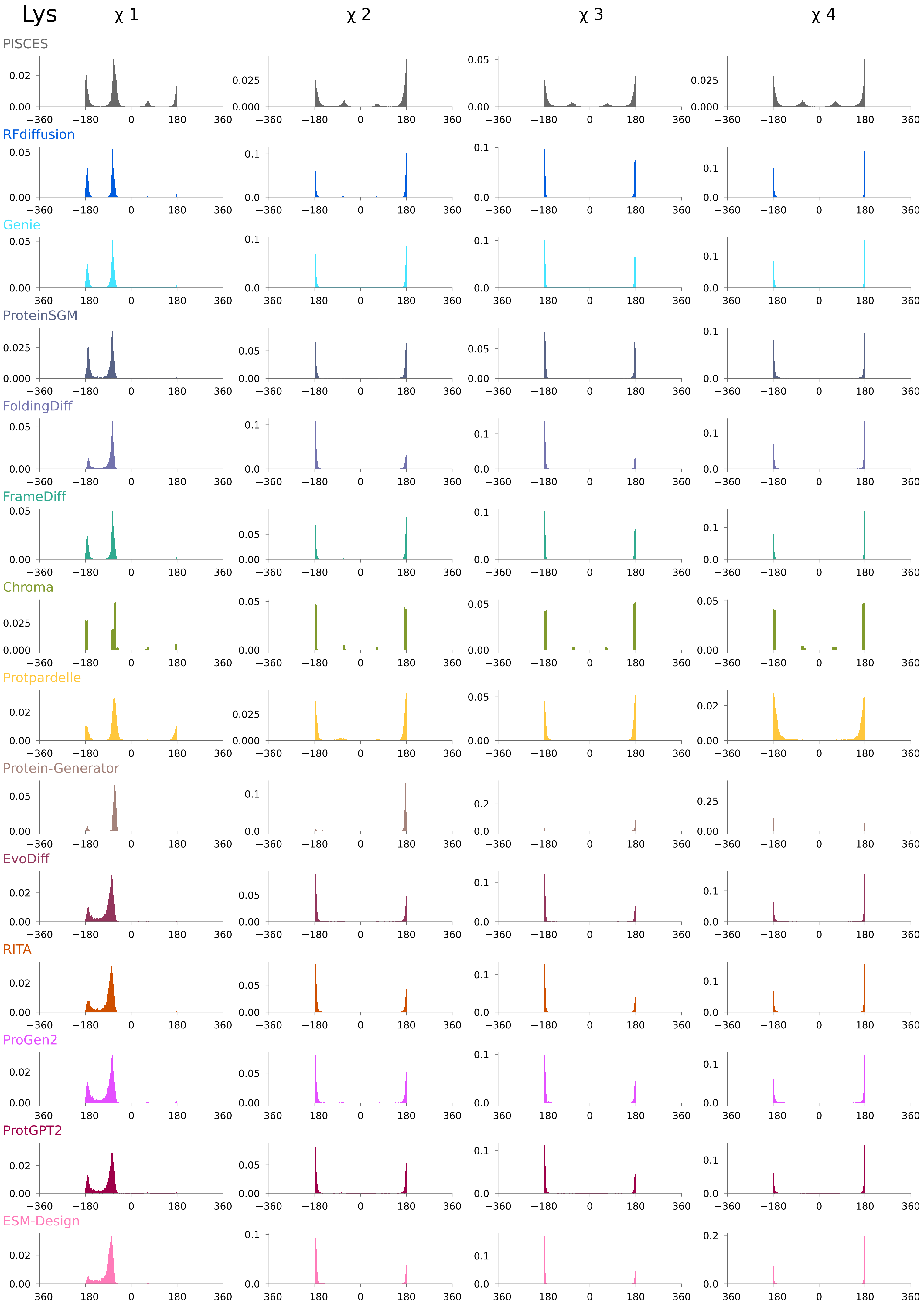
Lys
χ 1
χ 2
χ 3
χ 4
PISCES
RFdiffusion
Genie
ProteinSGM
FoldingDiff
FrameDiff
Chroma
Protpardelle
Protein-Generator
EvoDiff
RITA
ProGen2
ProtGPT2
ESM-Design

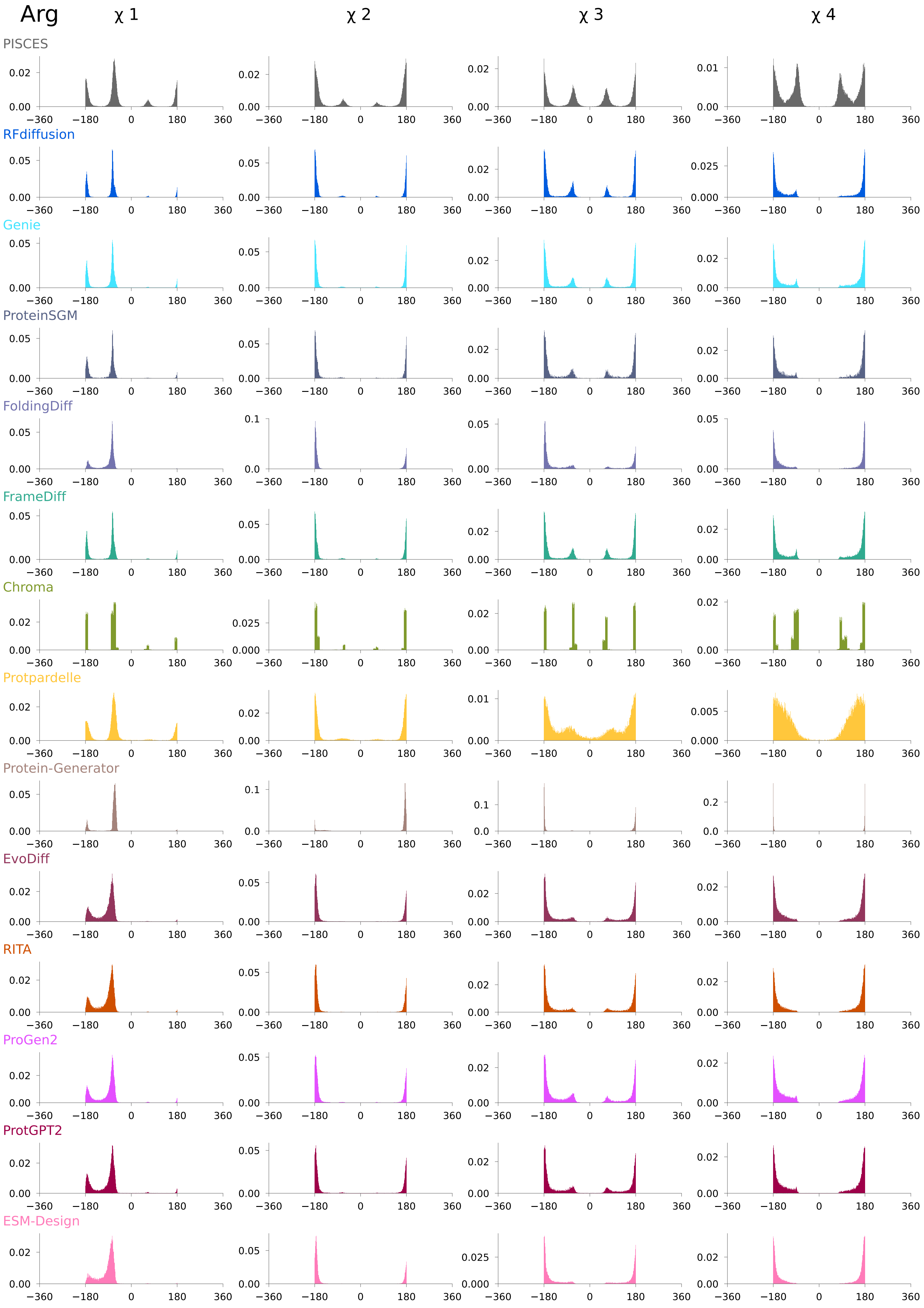
Arg
χ 1
χ 2
χ 3
χ 4
PISCES
RFdiffusion
Genie
ProteinSGM
FoldingDiff
FrameDiff
Chroma
Protpardelle
Protein-Generator
EvoDiff
RITA
ProGen2
ProtGPT2
ESM-Design
