# Supplementary material for: Benchmarking Generative AI Protein Models Reveals Differences Between Structural and Sequence-based Approaches
Source: Genomics Proteomics Bioinformatics. 2026 Feb 15;24(2):qzag014. doi: 10.1093/gpbjnl/qzag014 (PMC13472679; doi:10.1093/gpbjnl/qzag014)
Supplement: Supplementary file 1 [file qzag014_supplementary_data.zip › Figure S14.pdf]

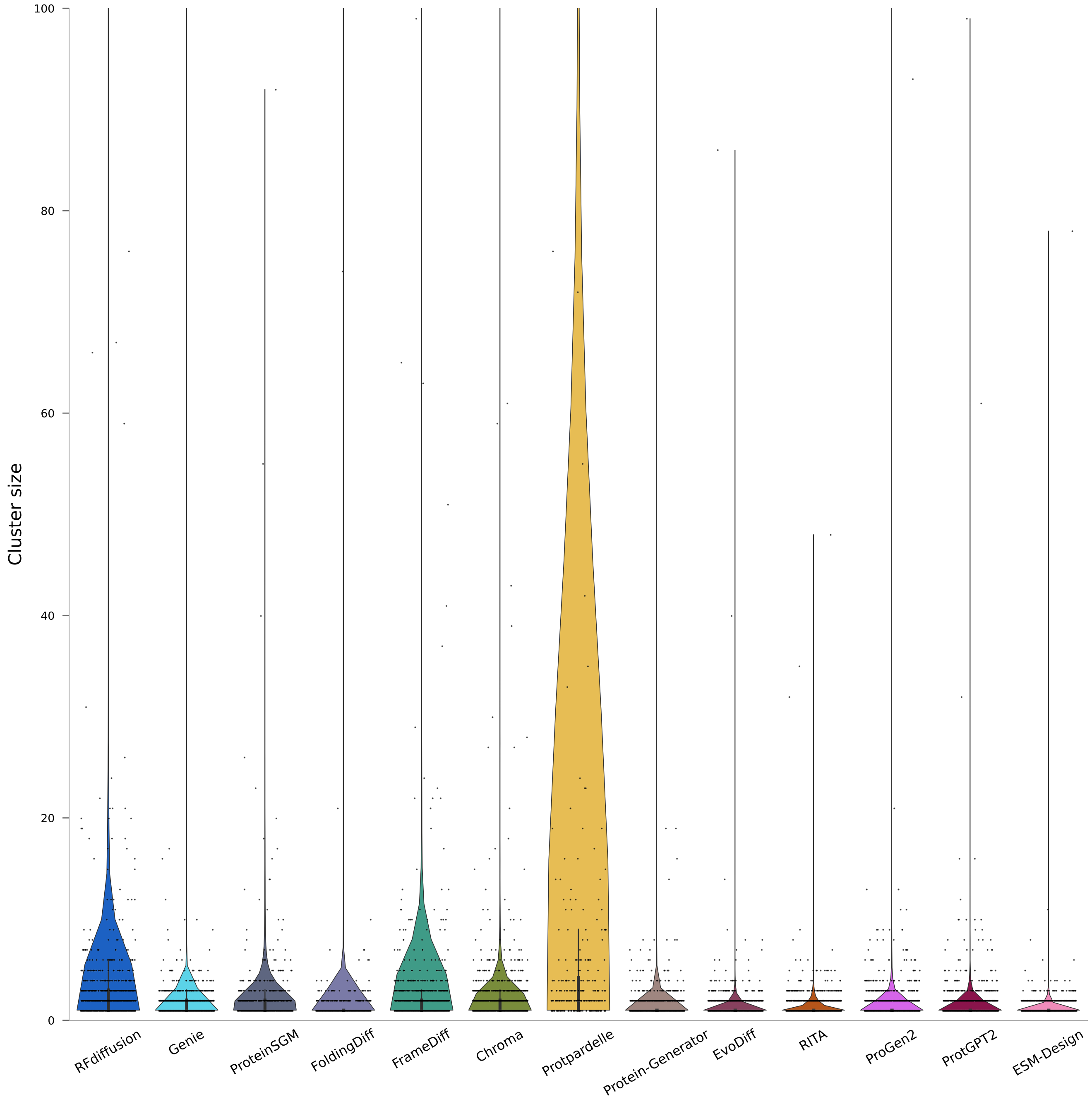

Cluster size
100
80
60
40
20
0
RFdiffusion
Genie
ProteinSGM
FoldingDiff
FrameDiff
Chroma
Protpardelle
Protein-Generator
EvoDiff
RITA
ProGen2
ProtGPT2
ESM-Design
